# Sheathless inertial particle focusing methods within microfluidic devices: a review

**DOI:** 10.3389/fbioe.2023.1331968

**Published:** 2024-01-08

**Authors:** Tao Peng, Jun Qiang, Shuai Yuan

**Affiliations:** ^1^ Zhuhai UM Science & Technology Research Institute, Zhuhai, China; ^2^ The School of Mechanical Engineering, Ningxia University, Yinchuan, Ningxia, China; ^3^ State Key Laboratory of High Performance Complex Manufacturing, College of Mechanical and Electrical Engineering, Central South University, Changsha, Hunan, China

**Keywords:** passive focusing, microfluidic device, inertial focusing, elasto-inertial focusing, sheathless focusing

## Abstract

The ability to manipulate and focus particles within microscale fluidic environments is crucial to advancing biological, chemical, and medical research. Precise and high-throughput particle focusing is an essential prerequisite for various applications, including cell counting, biomolecular detection, sample sorting, and enhancement of biosensor functionalities. Active and sheath-assisted focusing techniques offer accuracy but necessitate the introduction of external energy fields or additional sheath flows. In contrast, passive focusing methods exploit the inherent fluid dynamics in achieving high-throughput focusing without external actuation. This review analyzes the latest developments in strategies of sheathless inertial focusing, emphasizing inertial and elasto-inertial microfluidic focusing techniques from the channel structure classifications. These methodologies will serve as pivotal benchmarks for the broader application of microfluidic focusing technologies in biological sample manipulation. Then, prospects for future development are also predicted. This paper will assist in the understanding of the design of microfluidic particle focusing devices.

## 1 Introduction

Microfluidics allows precise manipulation of droplets, cells, and bacteria on a micro-scale (Whitesides, 2006). Compared with traditional micro-scale systems, microfluidics has cost-saving advantages, low energy consumption, and high efficiency (Nagrath et al., 2007). Microfluidics promises to be a revolutionary technology in biomedicine and clinical diagnostics due to the ability to precisely manipulate less volume of fluid. In recent years, microfluidics has been developed for many applications, such as focusing (Xuan et al., 2010), separation (Hettiarachchi et al., 2023), and trapping (Nilsson et al., 2009). Microfluidics has been widely used in chemical, biological, environmental, and other fields. Among these applications, particle focusing refers to aligning and arranging dispersed particles into ordered single or multiple trains in microfluidics, which is often a prerequisite step for downstream processing, such as enrichment, detection, separation, and manipulation of target particles, benefiting from the powerful and automatic arranging function (Zhang et al., 2020).

Classified according to the principle of manipulation, there are mainly three kinds of focusing methods relating to microfluidics: active, sheath-assisted, and passive focusing methods. Active methods rely on external forces such as acoustic (Rufo et al., 2022), magnetic (Liu et al., 2023), and optical (Yang et al., 2022) forces to control particle motion to achieve the goal of arranging particles into one or multiple ordered trains. Active focusing has higher flexibility and control accuracy. However, the additional external energy field requires sophisticated control systems and increases the complexity of microfluidic devices (Zhu and Wang, 2017). Often, the particle fluid is required to maintain a low flow rate to ensure that particles can be subjected to sufficient external force to move to the focusing equilibrium position, making it difficult to achieve high throughput.

Sheath-assisted focusing uses additional sheath flows to pinch particle flow into a narrow single train (Mao et al., 2007). Sheath-assisted focusing can achieve high throughput, but introducing an external fluid requires additional geometrical microchannels and micro-pumps for sheath inputs, which reduces the integration of microfluidic devices (Chiu et al., 2013). Among passive focusing techniques, inertial focusing utilizes the inertia of fluid (Zhang et al., 2016), viscoelasticity of non-Newtonian medium (Zhang et al., 2016; Lu et al., 2017), or micro-vortex induced by channel structure (Jiang et al., 2021) to control the transverse position of particles to achieve the focusing effect. The integration and footprint of microfluidic devices are effectively improved (Lim et al., 2014). When used in cell focusing, it can attenuate cell damage caused by the external fluid or force. This focusing method can achieve continuous, high-throughput, and label-free particle manipulation. The elasto-inertial focusing principle is generally distinguished due to the different fluid mediums. Among sheathless inertial focusing methods, the inertial focusing technique is generally 2D focusing, i.e., particles are arranged in a single train only in a 2D plane. To achieve 3D focusing, some measures can be taken, such as combining with different morphologies or changing channel structure; however, elasto-inertial focusing can achieve 3D focusing.

There are some scientific literature works with reviews of inertial microfluidics (Amini et al., 2014; Shi, 2023), non-Newtonian microfluidics (Yuan et al., 2018), sheath-assisted focusing (Lu et al., 2016), secondary flow in inertial microfluidics (Zhao et al., 2020), sub-micrometer particle focusing (Zhang et al., 2020), channel innovations for inertial microfluidics (Tang et al., 2020), passive microfluidic separation and sorting (Bayareh, 2020), passive microfluidic driving method (Narayanamurthy et al., 2020), and inertial microfluidic separation (Xu et al., 2021). This review summarizes polymeric particles’ sheathless inertial focusing in microfluidic devices. We clarified the focusing mechanism from theoretical analysis, simulation, and experimental perspectives. This review promises to provide insights into particle focusing and into the related biomedical applications.

## 2 Hydrodynamic forces and related principles

### 2.1 Hydrodynamic forces related to inertial microfluidic focusing

Inertial microfluidics relies on the intrinsic properties of fluids to achieve particle focusing. The forces related to the inertial effect are lift force induced by particle rotation (Magnus force, *F*_Ω_) (Rubinow and Keller, 1961), lift force induced by slip and shear motion of a particle (Saffman force, *F*_S_) (Saffman, 1965), lift force induced by the channel wall (*F*_WI_) (Zeng et al., 2005), and lift force induced by the shear gradient due to the curvature of the fluid velocity profile (*F*_SG_) (Matas et al., 2004). In most cases, Magnus and Saffman forces are usually neglected for being much smaller than *F*_WI_ and *F*_SG_. The Dean effect and viscoelastic effect will induce Dean drag force (*F*_D_) and elastic force (*F*_E_) in the microchannel, respectively. The forces can independently or synthetically affect the trajectory and equilibrium of particles to achieve different focusing states.

**FIGURE 1:**
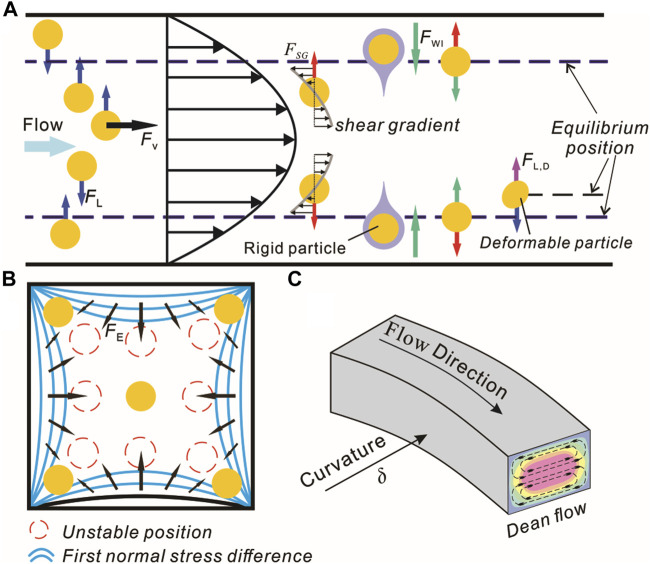
Schematic of hydrodynamic forces in microfluidics. **(A)** Inertial forces in a straight microchannel. **(B)** Elastic force distribution in a square cross-section microchannel. **(C)** Dean flow within a curved microchannel.

When the inertia of the fluid exists, the neutral buoyant particle will be subjected to the net inertial lift *F*_L_. *F*_L_ is the joint force of *F*_WI_ and *F*_SG_. The role of *F*_WI_ is to drive particles away from walls, and that of *F*_SG_ is to drive particles near the channel center to the walls. Under the action of joint force *F*_L_, particles will be stable at the equilibrium position between the channel center and the wall (Figure 1A). The expression of the *F*_L_ is given by (Di Carlo, 2009)

$$F_{L}=\frac{\rho_{f}U_{m}^{2}a^{4}}{D_{H}^{2}}f_{L}\left(R_{c},x_{c}\right),$$ (1)

where *ρ*_f_ is the fluid density, *U*_m_ is the maximum velocity of flow, *a* is the particle diameter, and *D*_H_ is the hydraulic diameter of the channel. For rectangular channels, *D*_H_ *= 2wh/*(*w + h*), where *w* and *h* are the channel width and height, respectively. *f*_L_ is the lift coefficient, which depends on the channel’s Reynolds number *R*_c_ and particle position *x*_c_ in a microchannel. The lift coefficient can be estimated via direct numerical simulation (Mashhadian and Shamloo, 2019), explicit formula (Su et al., 2023) or machine learning (Su et al., 2021).

The channel’s Reynolds number *R*_c_ and the particle’s Reynolds number (*R*_p_) are the dimensionless numbers related to the inertial effect. *R*_c_ describes the relationship between the inertial force and the viscous force. *R*_p_ introduces the ratio of particle diameter to channel hydraulic diameter.

$$R_{c}=\frac{\rho_{f}U_{m}D_{H}}{\mu },$$ (2)

$$R_{p}=R_{c}\frac{a^{2}}{D_{H}^{2}}=\frac{\rho_{f}U_{m}a^{2}}{\mu D_{H}},$$ (3)

where *μ* is the dynamic viscosity of the flow.

When the fluid medium changes from viscous to viscoelastic or Newtonian to non-Newtonian, the elastic force on particles should be considered (Feng et al., 2022). In pressure-driven viscoelastic flows, the first normal stress (*N*_*1*_ *= σ*_*xx*_ *-σ*_*yy*_) and the second normal stress (*N*_*2*_ *= σ*_*yy*_ *-σ*_*zz*_) will affect particle migration in the fluid. *σ*_*xx*_, *σ*_*yy*_, and *σ*_*zz*_ are normal stresses exerted in the flow and are directed toward the flow, velocity gradient, and vorticity direction, respectively. *N*_*2*_ is often negligible in the diluted viscoelastic solution for being much smaller than *N*_*1*_ (Pathak et al., 2004). The elastic force (Figure 1B) due to the non-uniform distribution of *N*_*1*_ acting on particles is (Leshansky et al., 2007)

$$F_{E}=C_{eL}a^{3}\nabla N_{1}={-2C}_{eL}a^{3}\eta_{p}\lambda \nabla {\dot{\gamma }}^{2},$$ (4)

where *C*_*eL*_ is the elastic lift coefficient and *η*_*p*_ is the polymeric contribution to the solution viscosity.

The dimensionless Weissenberg number *Wi* (Rodd et al., 2005; Rodd et al., 2007) is usually used to describe the viscoelasticity of a fluid and is defined as

$$Wi=\dot{\gamma }\lambda ,$$ (5)

where $\dot{\gamma }$ is the characteristic shear rate and _*λ*_ is the relaxation time of fluid. In a rectangular microchannel, $\dot{\gamma }=2U_{m}/h$ and $Wi=2\lambda Q/hw^{2}$ (Nam et al., 2012), where *Q* is the volumetric flow rate.

When flowing through a curved microchannel with a parabolic velocity distribution and flow inertia, two vortexes with opposite rotational directions perpendicular to the flow direction are generated, known as Dean secondary flows (Figure 1C). The intensity of the Dean flow can be expressed with the Dean number (Zhao et al., 2020):

$$De=R_{c}\sqrt{\frac{D_{H}}{2\delta }},$$ (6)

where *δ* is the curvature of the curved channel, and the Dean drag force acting on a particle can be expressed as

$$F_{D}=3\pi \mu aU_{D}.$$ (7)

The magnitude and direction are determined by the distribution of Dean flow in the cross-section. The velocity of the Dean secondary flow can be estimated with $U_{D}∼1.8\times 10^{-4}De^{1.63}$ (Ookawara et al., 2004).

In a uniform Stokes fluid, the viscous drag force on a rigid particle due to the difference between the particle and fluid velocity is (Yuan et al., 2018)

$$F_{v}=3\pi \mu a\left(U_{f}-U_{p}\right),$$ (8)

where *U*_f_ and *U*_p_ represent the velocity of the fluid and particle, respectively; the direction of the viscous drag force is along the mainstream (Figure 1A).

Unlike rigid particles, the shape change of the deformable particles will induce additional lift force. The dimensionless numbers used to define the relative deformation of droplets are Weber number ( $We=\rho_{f}U^{2}a/\sigma$ ), capillary number ( Ca=μUa/h ), and viscosity ratio ( $\lambda_{d}=\mu_{d}/\mu$ ), where *σ* is the surface tension and *μ*_*d*_ is the dynamic viscosity of the fluid inside the drop. Deformability-induced lift force will be dominant as *Ca* increases and *R*_*p*_ decreases (Amini et al., 2014). When a drop or bubble is not too close to the channel wall, the expression of deformability-induced force can be (Stan et al., 2013) $F_{L,D}=Ca\mu Ua{\left(\frac{a}{H}\right)}^{3}\left(\frac{d}{H}\right)f\left(\lambda_{d}\right)$ , where *d* is the distance between the droplet and the channel center and *U* is the characteristic velocity of the fluid. The equilibrium position of the deformable particles is usually farther from the wall compared to that of rigid particles (Figure 1A) (Hur et al., 2011). The above forces can be used to analyze particles’ focusing behavior in inertial microfluidic focusing devices.

### 2.2 Principles related to inertial focusing

In inertial microfluidics, particles’ focusing behavior must satisfy the confinement ratio and force ratio principles. These principles ensure that the particles are subjected to sufficient forces in the microfluidic channel to achieve equilibrium. In inertial focusing, a better-focusing effect can be achieved by meeting the requirements of confinement ratio *a/D*_H_ *>*0.07 (Kuntaegowdanahalli et al., 2009). By ensuring this condition, particles can have sufficient inertial force for migration to the equilibrium position. In elasto-inertial microfluidics, it has been proven that focusing can be realized under *a/D*_H_ *<0.04* (Young Kim et al., 2012; Lee et al., 2013) by using the spiral channel with extended length in a quite small footprint or a smaller channel size relative to the particle diameter with adequate elasticity.

The relative balance of forces on a particle determines the motion behavior in inertial focusing. In a curved channel, the focusing effect will be affected by the ratio of *F*_L_ */F*_D_ (Bhagat et al., 2008; Kuntaegowdanahalli et al., 2009). The ratio of *F*_L_ */F*_D_ can be scaled as

$$R_{f}=\frac{F_{L}}{F_{D}}∼\frac{1}{\delta }{\left(\frac{a}{D_{H}}\right)}^{3}R_{c}^{n},$$ (9)

where *f*_*L*_ scales with *R*_*c*_^*n*^ and *n* < 0. *R*_f_ needs to meet the following requirements when designing a curved microfluidic device for particle focusing (Amini et al., 2014). When *F*_D_ is much larger than *F*_L_, the inertial lift force can be neglected, and particles will rotate with Dean flow. When *F*_D_ and *F*_L_ are equal in magnitude, particle focusing will be achieved, and the equilibrium position can be controlled by adjusting the intensity of Dean flow (i.e., changing the curvature or flow rate). When *F*_D_ is much less than *F*_L_, the effect of particle focusing will be the same as inertial focusing in a straight channel.

In a non-Newtonian fluid medium, the elastic number *El*, which describes the elastic to inertial force ratio, will determine the focusing process. The expression of *El* is

$$El=\frac{Wi}{R_{c}}=\frac{\lambda \mu \left(w+h\right)}{\rho_{f}w^{2}h}.$$ (10)

*El ≈ 0* means that the elastic force can be negligible, and *El ≥ 1* means that the inertial force can be negligible. *El* is determined by the channel cross-section size and the characteristics of the fluid medium (Nam et al., 2012). When the fluid viscosity remains constant, *El* will be independent of the flow rate (Amini et al., 2014). The dynamic balance between forces can describe the particle-focusing mechanism. Equations 1-10 helps explain the mechanism and design of inertial microfluidic focusing chips.

## 3 Review on sheathless inertial microfluidic focusing technologies

**FIGURE 2:**
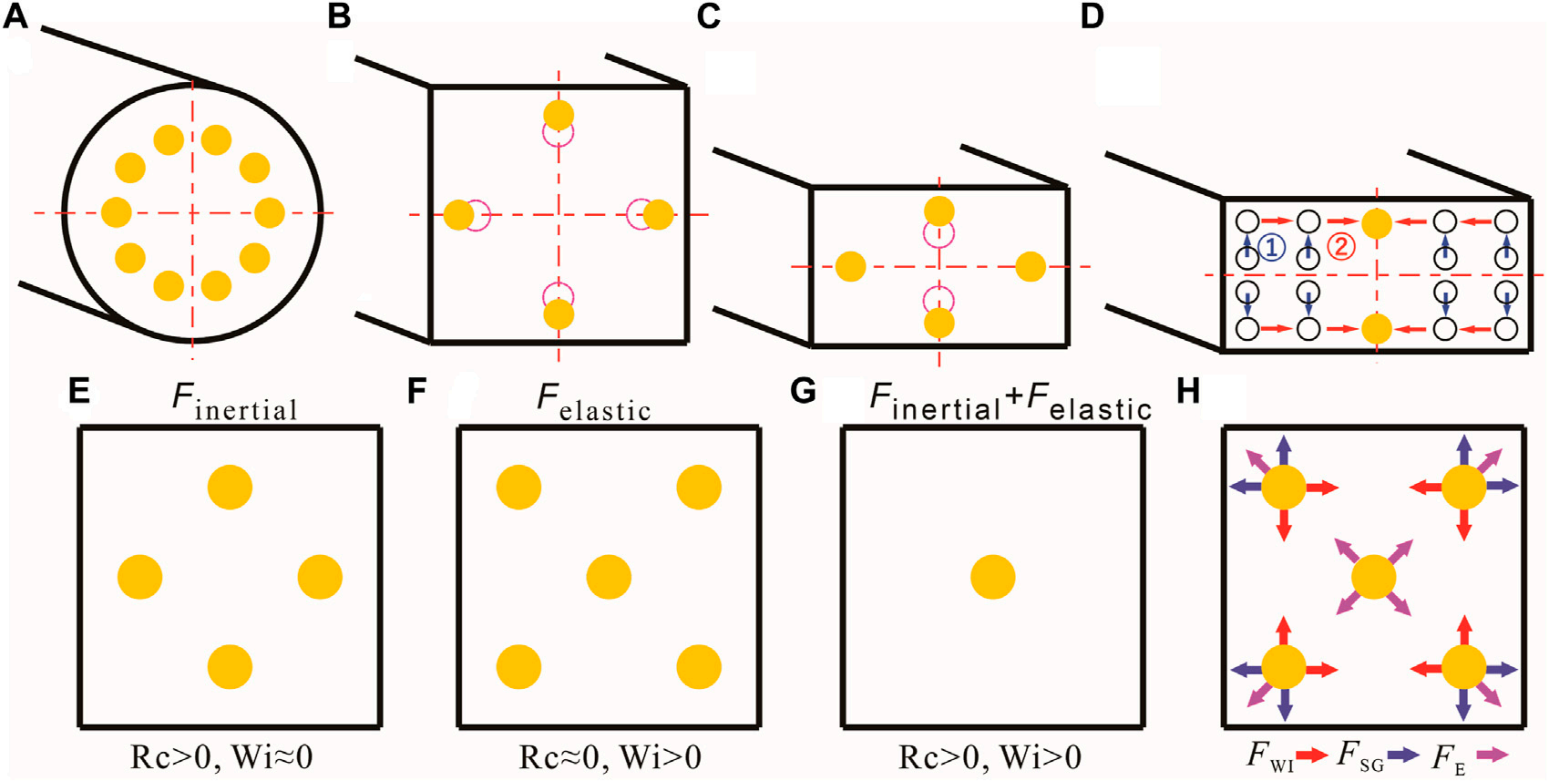
Inertial focusing of particles in the cross section. **(A)** In a circular cross-section pipeline, particles focus on 0.6 *R* (*R* is the pipe radius) at the exit. **(B)** Focusing position in a square channel (*AR* = 1); the dotted line represents the initial focus position, and the solid line represents the equilibrium position after the particle Reynolds number increases. **(C)** Equilibrium position in rectangular cross-section channels (*AR* < 1): as the Reynolds number increases, the equilibrium position changes from 2 to 4. **(D)** A two-stage migration model explaining inertial focusing mechanisms in a straight microchannel (Zhou and Papautsky, 2013). **(E–H)** Elasto-inertial focusing mechanisms in a square microchannel.

The inertial focusing method is widely used, independent of the external force field and other sophisticated auxiliary devices. Inertial focusing refers to controlling the lateral migration of particles by employing fluid inertia and eventually arranging particles into one or multiple trains. In 1961, Segré-Silberberg discovered that in a cylindrical pipe (∼1 cm in diameter), the neutral buoyant particles flowing in a laminar flow distribute equitably at the outlet from 0.6 times pipe radius to the center of the pipe (Figure 2A) (Segré and Silberberg, 1961). This effect is due to the inertial migration caused by the inertial lift force acting on the particles. Since then, the research field of particle inertial focusing has been pioneered. Inertial focusing was first applied to microfluidic applications in 2007 (Di Carlo et al., 2007). When introducing viscoelastic fluid into a circular microchannel, the randomly distributed particles focus on the central lines and disperse as the flow rate increases due to the high *F*_*SG*_ directed to the wall and the obvious shear-thinning effect (Seo et al., 2014). This section reviews inertial focusing in microfluidic devices from the channel configuration aspects.

### 3.1 Particle focusing in straight microchannels

Straight microchannels have been widely used in microfluidic applications for their simple structure and easy manufacturing. Among straight microchannels, the most frequently used are square [*AR* = 1 (aspect ratio = channel height/width) and rectangular cross-section channels (*AR* ≠ 1)]. In a rectangular channel, the net lift *F*_L_ balancing Stokes drag force shifts the particle to equilibrium. When *R*_p_ *<<* 1, the viscous interaction of the fluid and particles dominates particle motion. Under this condition, particles are subjected to the dominant surface drag and move to follow fluid streamlines. As *R*_p_ increases to 1, inertial forces dominate, and lateral migration across fluid streamlines becomes obvious (Di Carlo et al., 2007), forming a focusing phenomenon. In a square channel, the randomly distributed particles focus on four equilibrium positions along the center of each channel wall (Figure 2B) and then move toward the wall as *R*_*p*_ increases due to the strengthening of *F*_SG_ (Di Carlo et al., 2007).

The focusing properties in the straight microchannel can be affected by aspect ratio, cross-section morphology, particle confinement ratio, and flow rate. In the rectangular channel (*AR* ≠ 1), due to the blunted velocity profile along the wider surface, particles focus on two equilibrium positions along the center of the longer surface (Ciftlik et al., 2013). Notably, with an increase in the flow rate, the two unstable equilibrium positions along the short-channel side reappear, with *F*_WI_ becoming less dominant than *F*_SG_, and the equilibrium positions recover from two to four (Figure 2C). The detailed inertial focusing principle in the straight microchannel was explained through a two-stage focusing model (Figure 2D) (Zhou and Papautsky, 2013). In a low-*AR* rectangular microchannel, particles move to an equilibrium position where *F*_WI_ and *F*_SG_ are balanced. Then, particles near the wall move toward the wall-centered equilibrium position under the rotation-induced force *F*_*Ω*_. Particles moving to the first-stage equilibrium usually take less time than those moving to the second stage. The model can also explain the principle of inertial focusing under different cross-section microchannels.

The negligible elastic effect in viscoelastic fluids can affect and regulate particle migration behavior. The particle-focusing process depends on the rheological properties of fluids and the first principal stress difference (Caserta et al., 2011; Bai et al., 2023). The shear viscosity of viscoelastic fluids is generally divided into the constant viscosity region and the shear-thinning region. Particle focusing usually occurs in the constant viscosity region. The elasto-inertial focusing concept was first proposed by Yang et al. (2011) to illustrate 3D focusing in a straight square channel. When particles flow in a viscoelastic fluid with finite inertia, the particle focusing becomes intriguing, and single-train focusing will be achieved under the inertial and elastic effects.

**FIGURE 3:**
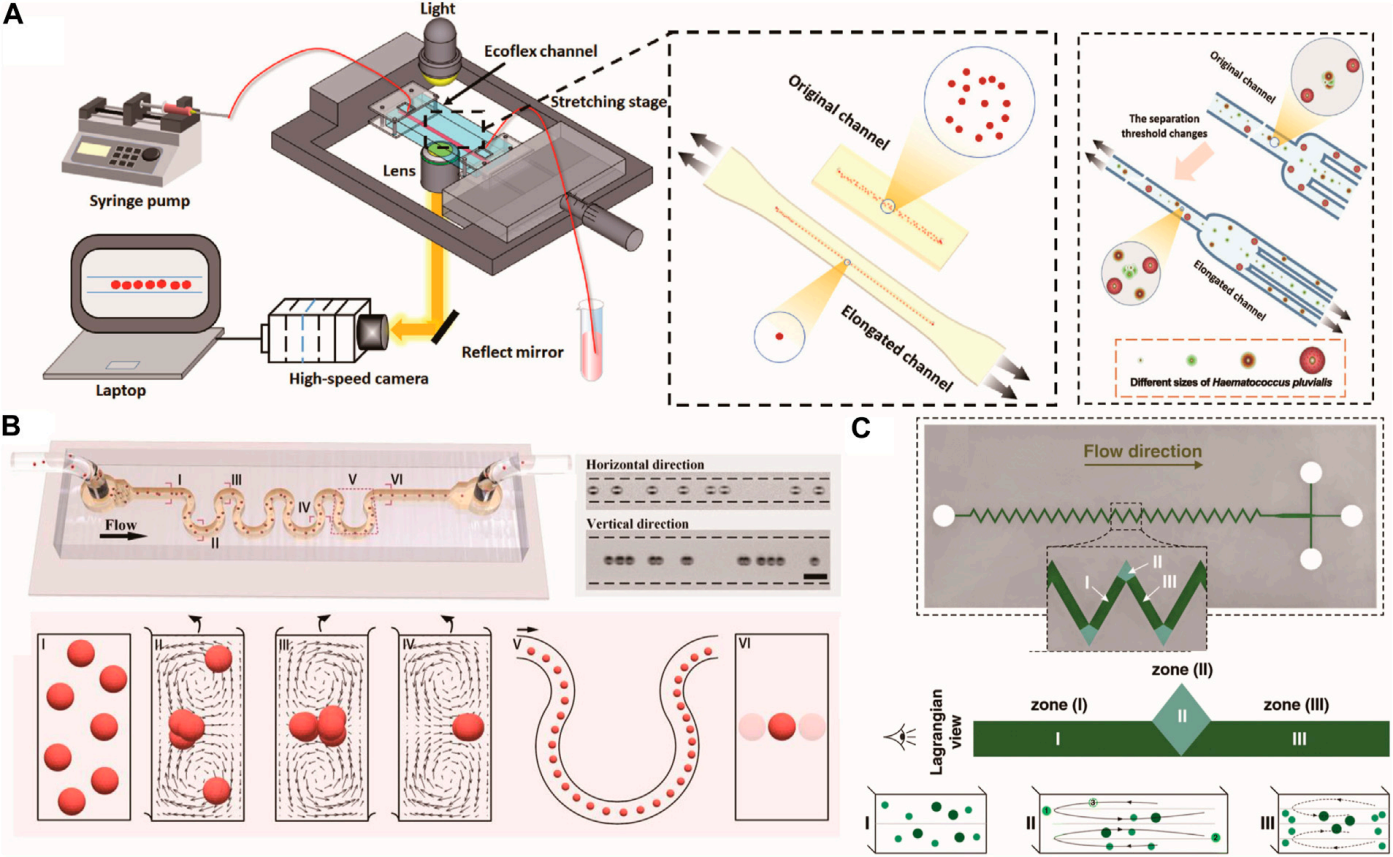
Particle focusing regulation in inertial microfluidics. **(A)** Ultra stretchable microfluidic platform for enhancing particle focusing in the straight microchannel (Jia et al., 2023). Copyright 2023, American Chemical Society. **(B)** 3D single-line inertial focusing in the high aspect ratio serpentine microchannel (Ni et al., 2022). Copyright 2023, American Chemical Society. **(C)** Particle focusing in the zigzag serpentine microchannel. Reproduced from Razavi Bazaz et al. (2022) with permission from the Royal Society of Chemistry.

In a square microchannel with a viscoelastic fluid, randomly distributed particles focus on four equilibrium positions near each center of the channel wall due to the inertial effect only (*Re > 0* and *Wi = 0*) (Figure 2E) (Zhou and Papautsky, 2020). When subjected to elastic forces only (*Re ≈ 0* and *Wi > 0*), particles focus on five equilibrium positions, with four near the corner and one at the central line (Figure 2F) (Yang et al., 2011; Liu et al., 2015). When elastic and inertial effects exist simultaneously (*Re > 0* and *Wi > 0*), under a moderate *El* number, 3D focusing with high precision can be achieved at the centerline (Figure 2G) (Yang et al., 2011; Del Giudice et al., 2013). In viscoelastic microfluidics, the 3D single-train focusing can only be achieved with the synergistic effects of inertia and elasticity (Figure 2H). The equilibrium position at the corner caused by elasticity can be easily destroyed by inertial force. In contrast, the equilibrium position at the central line is maintained with elastic force (Yang et al., 2011). The dimensionless numbers related to elasto-inertial focusing are elasticity number (*El*), Reynolds number (*R*_c_), and Weissenberg number (*Wi*), among which *Wi* is the most adequate one for evaluating the focusing efficiency (Song et al., 2016). In a low *AR* channel, the difference in first principal stress is more evident along the height direction than the width direction. The larger elastic force drives particles to the centerline of width. The lateral migration is mainly determined by inertial force because of the weakened elastic effect near the narrow surface (Won Seo et al., 2014). Multiple focusing trains were observed in low *AR* channels (Yang et al., 2017). In a square channel, by increasing the corner angle, the equilibrium position at the corner can also be reduced, and a more accurate single-train focusing can be obtained (Raoufi et al., 2019). Jia et al. (2023) developed a size-tunable elasto-inertial separation microfluidic system using an ultra-stretchable channel (Figure 3A). Channel stretching alters the local flow velocity distribution and strongly regulates particle focusing.

Inertial focusing in a straight microchannel is usually more suitable for larger micron-sized particles. Sub-micron particles (0.1–1 μm) generally require longer channel length or smaller cross-section (*F*_L_ *∼ D*_H_^*−2*^) (Bhagat et al., 2009; Zhou and Papautsky, 2013) for the net lift force scales with particle diameter *F*_L_ *∼ a* ^*4*^ (Guan et al., 2013), increasing manufacturing and detection complexity.

With viscoelastic fluids, particles with a diameter down to 200 nm can be focused remarkably (Leshansky et al., 2007). The focusing behavior of the submicron particles is determined by elastic force, inertial force, and Brownian motion force (Young Kim et al., 2012). Effective focusing of particles of diameter less than 100 nm can be achieved using oscillatory viscoelastic microfluidics, eliminating the need for long microchannels (Mutlu et al., 2018; Asghari et al., 2019).

Elasto-inertial focusing can also be used for deformable particles, which are subjected to additional lift induced by the channel wall due to deformation. The focusing method can be applied to particle separation due to different equilibrium positions and deformability (Yang et al., 2012). In addition to the widely used PEO and PVP viscoelastic solutions, other solutions are also used as viscoelastic fluids. Lim et al. (2014) reported extremely high-throughput focusing (*Q* = 50 ml/min and *Re* ∼10,000) in the hyaluronic acid (HA) solution with an epoxy microchannel, which demonstrated the highest flow rate yet achieved. Kim and Kim (2016) investigated the particle focusing process in a diluted *λ*-DNA solution. The throughput can be enhanced significantly due to the long relaxation time of DNA and high elasticity.

For straight channels with different cross-section shapes, particles will have dissimilar focusing effects for diverse velocity gradient profiles compared to a rectangular cross-section. By processing the master die with micro-milling and 3D-printing methods, various cross-sections, such as trapezoidal, triangular, and semi-circular, can be manufactured. Under the asymmetric axial velocity profile, the equilibrium positions will be derived to the long trapezoidal side between the centerline and the bottom when the trapezoidal cross-section is used. The focusing equilibrium will change as the angle of the slanted wall increases. This focusing method can also be applied to filtration of larger particles (Moloudi et al., 2018). Compared to a trapezoid cross-section, the triangular cross section makes realizing a single-streamline focusing near the channel apexeasier, especially with a low *AR* channel (with a 120° apex angle, *AR* = 0.4) (Mukherjee et al., 2019). The number of equilibrium positions will increase as the apex angle reduces. In a semicircular cross-section microchannel, particles are focused near the top and bottom of the centerline, and the position changes are unobvious with *R*_p_ altered (Kim et al., 2016). Kim U. et al. (2022) compared and investigated the particle behavior in rhombic and equilateral hexagonal cross-section microchannels with different flow rates and particle sizes, and this microfluidic device with non-rectangular microchannels can be used for bioparticle sorting.

### 3.2 Particle focusing in curved microchannels

Straight channels have the limitations of slow lateral migration, considerable resistance, and multiple equilibrium positions. Secondary flow (perpendicular to the main flow) within a curved channel will overcome these problems (Tang et al., 2020). Meanwhile, curved channels can effectively reduce the length needed for focusing, accelerate the focusing process, reduce focusing positions, and improve the integration of microfluidic devices (Wang and Dandy, 2017). This section reviewed the sheathless inertial particle focusing techniques from channel aspects: serpentine, spiral, and other curved channels.

#### 3.2.1 Focusing in the serpentine microchannels

Di Carlo et al. (2007) first studied the particle-focusing process in a serpentine channel. Introducing a symmetric or asymmetric curvature can reduce the four equilibrium positions in a straight, square channel to one or two, depending on the flow rate. This focusing mechanism can be applied for particle separation and filtration based on the different equilibrium positions (Di Carlo et al., 2008; Wang et al., 2018).

When used for submicron-sized particle focusing, smaller channel sizes and larger flow rates are required to ensure adequate lateral migration force. Wang and Dandy (2017) manufactured an asymmetric serpentine channel with the thermoset polyester (TPE) casting method and demonstrated single-stream focusing of submicron particles through a controllable flow rate. TPE has an elastic modulus that is 1,000 greater than that of polydimethylsiloxane (PDMS), with a favorable stiffness ratio of 1:10, which is suitable for microfluidic applications requiring high load-bearing pressure. This focusing method pushes the inertial focusing particle range down to the sub-micrometer level and can be applied for micron particle separation from the micro-nanoparticle mixture (Wang and Dandy, 2017).

Paiè et al. (2017) manufactured 3D microfluidic loops with femtosecond laser irradiation followed by chemical etching. The use of 3D loops produces a strong Dean flow with low resistance, which can be used for compact parallelization of multiple focusing. Zhou et al. (2018) adopted a series of reversed wavy channel structures generating periodically reversed Dean flow perpendicular to direction of the main flow to focus particles. As the *R*_*c*_ increases, the alternation in the Dean flow direction in the serpentine channel produces special hydrodynamic effects to focus particles. Ni et al. (2022) developed high aspect ratio asymmetric serpentine (HARAS) microchannels for single-line inertial focusing of particles and cells at the 3D center of the channel and observed size-independent and positionally controllable focusing phenomena over a wide range of flow rates (Figure 3B).

For symmetric serpentine microchannels, Zhang et al. (2019) systematically studied the effects of viscosity, flow conditions, particle size, and cross-section dimensions on focusing effects. Focusing patterns can be classified as two-train focusing, single-train focusing, and defocusing, which depend on the different focusing parameters. A parametric map of $F_{L}/F_{D}=\left(a/D_{H}\right)/\left[{D_{e}}^{1/2}{\left(D_{H}/2\delta \right)}^{3/4}\right]$ related to $\left(a/D_{H}\right)\times 2/\left(1+h/w\right)$ was summarized (Zhang et al., 2019), which can be used for predicting the focusing behavior of particles with specific sizes and guiding the structure design of microfluidic devices. Özbey et al. (2016) experimentally decoupled the effects of inertial forces and Dean drag force in a multi-curvature symmetric serpentine channel, which gives an insight into the underlying migration mechanism.

Yuan et al. (2019) studied the distribution of particles and cells in viscoelastic fluids, where inertia becomes dominant and destroys the focusing of particles above a certain flow rate. Instead of using a syringe pump, Shamloo and Mashhadian (2018) performed the simulation by using a centrifugal platform to drive fluid. The focusing behavior of particles is a combination of Dean drag force, inertial lift force, and centrifugal force. The channel length required for focusing is related to particle density and channel corner angles to the driving method. Removing pumps from centrifugal platforms provides an efficient integration for microfluidic devices. Zhang et al. (2014) adopted a low *AR* right-angled transitional serpentine microchannel based on the Dean drag and centrifugal force to focus particles. The inertial lift force is weakened due to the blunted velocity profile along the direction of the wider surface. The number of focusing trains is related to the particle size and channel Reynolds number. Single-train focusing can be achieved at the center of the long surface at a moderate Reynolds number. Amani et al. (2022) studied the inertial focusing behavior in the divergent serpentine microchannel, which showed an excellent performance compared to the convergent geometry in focusing (Razavi Bazaz et al., 2022; Ebrahimi et al., 2023) and developed a zigzag microfluidics channel that demonstrated superior separation and purity efficiency due to the sudden channel cross-section expansion at the corners compared to other serpentine microchannels, and the threshold of the particle-confinement ratio decreased to 0.04 (Figure 3C).

In serpentine microchannels, particle focusing accuracy can be regulated by channel expansion or the introduction of micro-obstructions. Lu et al. (2020) proposed a microfluidic chip introducing periodic micro-square contractions to the serpentine channel, which provides a strategy for developing high-precision inertial sorting. The transition region shrank to the sub-micron scale, and the separation of 6.0 and 5.5 μm particles with a higher recovery ratio was achieved (Lu et al., 2020). Cha et al. found that embedding asymmetric obstacle microstructures in a symmetric serpentine flow channel adjusts inertial focusing and reduces the equilibrium position. The asymmetric concave obstacle destroys the symmetry of the original inertial focusing position, which leads to unilateral focusing (Cha et al., 2022; Cha et al., 2023b).

#### 3.2.2 Focusing in Archimedes’ spiral microfluidic microchannels

Archimedes spiral is commonly used in microfluidic applications, benefiting from the compact structure and additional Dean drag force. With the increasing Dean number, the focusing process can accelerate, and particles can find the transverse stable position more quickly. Particles of different sizes have different *F*_L_ */F*_D_ values and will focus on the position near the inner wall according to size (Kuntaegowdanahalli et al., 2009). A spiral microchannel with increasing width and curvature can provide an additional secondary flow field for focusing. The particle will focus on two equilibrium positions along the height direction near the inner wall (Russom et al., 2009). Martel and Toner (2012) studied the focusing effect in spiral channels of different aspect ratios. As *AR* decreases, *F*_L_ along the wide surface decreases due to the blunted velocity profile, and the focusing process will be dominated by *F*_D_, which means that the equilibrium position can be obtained by regulating the flow rate. Jeon presented a mass-producible, clogging-free plastic spiral inertial microfluidic system, a material conversion from PDMS to harder plastics that will be crucial in realizing the vision of high-throughput industrial applications of inertial microfluidics (Jeon et al., 2022).

Establishing the corresponding model relates to the range of flow rate change and the focusing position, which will help elucidate the mechanism of the focusing process. Xiang et al. used a five-stage model to illustrate the focusing mechanism of the changing flow rate (Xiang et al., 2013b; Xiang et al., 2013a). As the flow rate increases, focusing patterns will change as follows: in the case of a low flow rate with non-negligible inertia and weakened Dean effect, particles focus along the center of the upper and lower wider surface. As the flow rate increases, the transversal motion will be dominated by *F*_L_, and particles will move toward the inner wall. The equilibrium position will be stable on the inner wall. Then, *F*_D_ exceeds *F*_L,_ and the equilibrium position moves toward the outer wall. Ultimately, defocusing and mixing effects were formed due to the faster strengthening of the Dean effect (Kuntaegowdanahalli et al., 2009). Xiang et al. (2015) investigated the migration dynamics of particles with different confinement ratios and summarized it with three modes: non-focusing, rough focusing, and distinct focusing. The work provides an insightful understanding of the focusing dynamics of particles.

**FIGURE 4:**
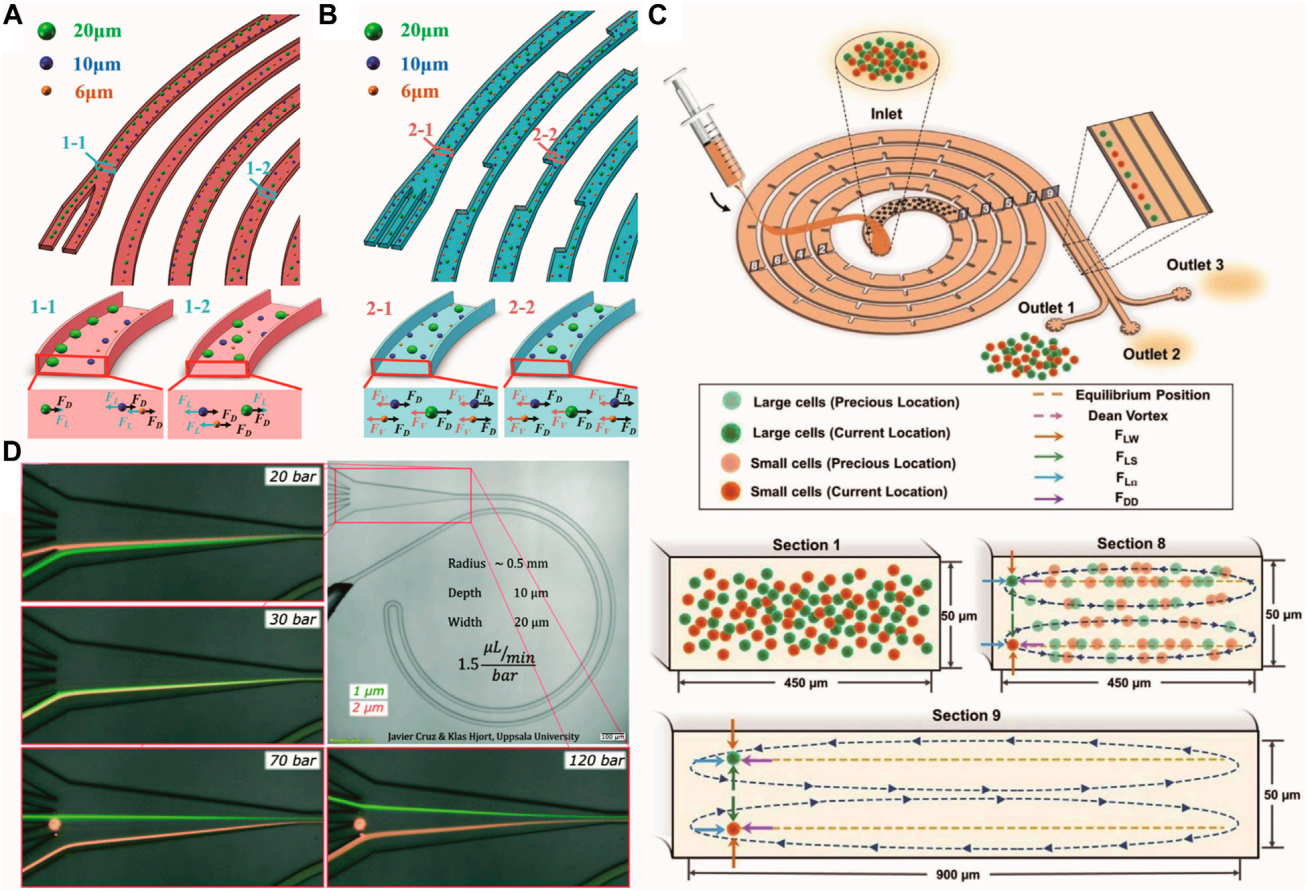
Particle focusing in the spiral microchannel. Comparison of the particle motions in the normal **(A,B)** Periodic expansion spiral channel (Gou et al., 2020). Copyright 2020, American Chemical Society. **(C)** Inertial focusing in the dimension-confined ultra-low aspect ratio spiral microchannel (Zhao et al., 2022). **(D)** Inertial focusing of submicron particles. Reproduced from Cruz et al. (2019) with permission from the Royal Society of Chemistry.

Guan et al. (2013) investigated the particle focusing process in a trapezoidal cross-section spiral microchannel. Strong Dean flow will be generated near the wider sidewall, which speeds up the trapping of particles toward the narrow side and accelerates the focusing process (Guan et al., 2013). Zhu et al. (2021) presented a polymer-film inertial microfluidic jigsaw sorter with trapezoidal spiral channels by accurately assembling polymer-film jigsaws of different thicknesses, which brings advantages to the flexible design of inertial microfluidics. The Dean effect can be controlled and enhanced by increasing the slant angle. The acceleration of Dean flow intensity can also be realized by introducing micro-obstacles into the spiral microchannel, as reported by Shen et al. (2017). The focusing performance will be improved, and the focusing process will speed up. The manufactured device can be promising for the application of high-throughput microfluidics systems. However, the introduced micro-obstacles will increase the flow resistance and require higher bonding strength for microfluidic chips. Accurate single-train focusing can also be obtained in spiral microfluidics by adopting an appropriate channel contraction (Gou et al., 2020) or arranging obstacles (Cha et al., 2023a). Introducing asymmetric periodic expansion structures in the spiral channel produces a stable additional vortex-induced lift force, combined with Dean drag force, effectively enhancing the focusing process (Gou et al., 2020) (Figures 4A, B). Inertial microfluidic chip design usually requires flow rate optimization, which increases the development cycle. By introducing a series of microstructures in a dimension-confined ultra-low aspect ratio spiral microchannel, Zhao et al. (2022) demonstrated flow-rate and particle-size insensitive inertial focusing of 15.5-μm particles and tumor cells, which will be promising for cytometry application (Figure 4C).

In a spiral microchannel with viscoelastic fluid, the equilibrium positions are determined by the ratio of *F*_D_ to *F*_E_, which is proportional to *a* ^*2*^. Unlike the principle of inertial focusing, larger particles are focused close to the outer wall due to the existence of *F*_D_ and *F*_E_ (Kumar et al., 2021). This focusing mechanism can be used for *a/D*_H_ *< 0.04*, which makes up for the deficiency of inertial focusing. Xiang et al. (2016b) systematically studied the mechanism of elasto-inertial focusing in a spiral microchannel with different flow rates, aspect ratios, and curvatures. A six-stage model was proposed to explain the mechanism under different flow rates. The equilibrium position of particles is more sensitive to the flow rate change (Xiang et al., 2016b). Gao et al. (2023) systematically investigated particle elasto-inertial migration in spiral channels, which helped improve the understanding of the underlying mechanisms. When the fluid changes from water to PEO and then to PVP, the elasticity increases, and particles focus on the centerline due to excessive elastic force, which overweighs the inertial lift and Dean drag force (Xiang et al., 2016a). As the concentration of viscoelastic decreases, the position of the focusing equilibrium moves toward the outer wall under the combined influence of *F*_E_, *F*_D_, and *F*_L_ (Xiang et al., 2018).

#### 3.2.3 Focusing in other types of curved microchannels

In curved channels, inertial lift forces determine the focusing of particles, and the Dean flow is responsible for modifying the focusing equilibrium positions. Under different Dean number conditions, lateral migration of particles to stable and unstable states will be caused. Increasing the radius of curvature or decreasing the hydraulic diameter will improve the single-train focusing. Gossett and Di Carlo (2009) investigated the effect of the degree of asymmetry on the particle-focusing process. With increased asymmetry, particles can quickly focus on a single train at lower *R*_c_. Martel and Toner (2013) decoupled the effects of *R*_c_ and *De* on the inertial focusing behavior and concluded that the focusing mechanism is more complex than an explanation of force balance (*F*_L_ */F*_D_). The equilibrium position of three particle sizes under critical parameters such as *R*_c_, confinement ratio, and curvature ratio was analyzed. Increasing curvature not only increases Dean drag force but also changes shear gradient lift force through redistribution of the velocity profile, which leads to a new vertical equilibrium position of particles (Martel and Toner, 2013). The detailed results can provide a guide for designing curved microfluidics channels.

By reducing the cross-section size to 20 × 10 μm^2^, Cruz et al. (2019) demonstrated single-line inertial focusing of spherical particles as small as 0.5 μm in a curved microchannel and extended it to bacterial sorting (Figure 4D). They explained the equilibrium and migration mechanisms through numerical simulations and experimental studies. High-aspect-ratio curved microchannels can achieve a single-focus position near the inner center, largely independent of the flow rate and separation at the submicron scale. In curved triangular channels, the inertial focusing could be easily tuned to achieve a single stream, which enables finding the best design for a specific application with less time and effort (Kim et al., 2022). More detailed viscoelastic microfluidic focusing within curved microchannels can be found in Feng et al. (2022). Table 1 lists particle focusing in microfluidics with Newton fluid, and detailed parameters are provided.

**TABLE 1:** Early observations of particle focusing with Newtonian fluid in microfluidic devices.

Structure	Microchannel	Dimension	Particle (*a* in μm)	Length fraction	Dimensionless number	Reference
Straight	Rectangular	(100, 120, 140, 160) × 25 μm^2^	PS (9.9)	0.06–0.93% v/v	R_p_ (0.4–1.6)	Reece and Oakeya (2016)
	Trapezoid	(100–200) × (50–70) μm^2^	PS (10, 20, 45)	0.1% v/v	R_c_ (20–800)	Moloudi et al. (2018)
	Rectangular	(25–35) ×47 μm^2^	PS (3, 6)	0.08% w/v	R_c_ (13–72)	Masaeli et al. (2012)
	Triangle	100 × 50 μm,	PS (7, 10, 15, 18)	∼1–2×10^5^ particles/mL	R_c_ (8.4–190)	Mukherjee et al. (2019)
		Apex angle 120°				
Serpentine	Symmetric serpentine	200 × 40 μm^2^	PS (8, 9.9, 13)	0.025%–0.1% w/w	R_c_ (118–200)	Zhang et al. (2014)
	Asymmetric serpentine	20 × 10 μm^2^ (min.)	PS (0.92, 2)	0.01–1% v/v	R_c_ (11.1–1,550)	Wang and Dandy (2017)
	Rectangular	I: 100 × 5 (min.) μm^2^	PS (10.2)	0.1% w/v	R_p_ (0.2–6.0)	Oakey et al. (2010)
		II: 30 × 50 μm^2^				
	Reverse wavy	125 × 40 μm^2^	PS (1, 3, 5, 7, 10, 15)	6×10^6^ particles/mL	R_c_ (10–40)	Zhou et al. (2018)
Multi-stage	Triangle + semicircle	W (50,80) H (40)	PS (7, 9.9)	0.05%–0.1% w/w	R_p_ (0.008–3.2)	Kim et al. (2016)
		Dia (50) H 40				
	Rectangular	50× (10, 25) μm^2^	PS (10, 15, 20)	0.1 % v/v	R_c_ (2.6–78)	Wang et al. (2015)
	Semicircular	69 × 92 μm^2^	PS (5, 7.32, 10.4,	∼105 particles/mL	∼	Zhang et al. (2018)
			15.45, 20.3)			
Spiral	Rectangular	250 × 50 μm^2^	PS (2, 7, 10)	0.1% w/v	De (0–30)	Russom et al. (2009)
	Rectangular	150 × 50 μm^2^	PS (5, 10)	0.5 wt%	De (0.86–15.53)	Xiang et al. (2013b)
	Rectangular	500 × 130 μm^2^	PS (10, 15, 20)	0.1%–0.3% v/v	De (4.4–14.6)	Kuntaegowdanahalli et al. (2009)
	With micro-obstacles	900 × 100 μm^2^	PS (7.3, 9.9, 15.5)	∼	Re (0–666.7)	Shen et al. (2017)
	Rectangular	100 × 50 μm^2^	PS (4.4, 9.9, 15)	0.021–0.23% v/v	R_c_ (0–400)	Martel and Toner (2013)
Micro-vortex	Stepped straight	81× (21, 41.5) μm^2^	PS (7.9, 9.9, 13)	0.1% w/w	R_c_∼ 83.33	Chung et al. (2013a)
	Rectangular expansion	(200, 40) × 50 μm^2^	PS (7)	0.25% v/v	R_p_ (0.8–3.5)	Park et al. (2009)
	Triangular expansion	50 × 70 μm^2^	PS (3.2, 4.8, 9.9)	∼10^5^ particles/mL	R_c_ (20.82–329.12)	Zhang et al. (2013)
	Sharp corner	80 × 50 μm^2^	PS (9.9)	3.5–5.5 × 10^3^ particles/μL	R_p_ (3.89–27.22)	Fan et al. (2014)
	Stepped straight	200 × 40 μm^2^	PS (9.9, 13, 24)	0.5–2 × 10^5^ particles/mL	R_c_ (29.2–218.7)	Zhao et al. (2017)

### 3.3 Micro-vortex-induced particle focusing

Micro-vortex refers to the emergence of a vortex in a fluid that can provide a helpful transversal drag force for particle migration. The vortex can be generated by gradually adjusting the cross-section area along the flow direction, the topological expansion or contraction of the straight channel, or the arrangement of micro-obstacles in a channel. There are usually two types of micro-vortexes: vertical vortex, perpendicular to the flow direction, and horizontal vortex, parallel to the flow direction. The balance between the drag force induced by the vortex and the inertial lift force can explain the focusing mechanism.

Choi and Park (2007) investigated the particle focusing process in microfluidics with slanted obstacles. The arrangement of slanted rectangular obstacles with a fixed angle in the microchannel can induce a vertical vortex, and the focusing operation can be achieved under the different fluid pressure distributions compared to a straight rectangular microchannel. The focusing method has a great relationship with particle size and relates slightly to the flow rate (Choi and Park, 2007). The particle-focusing process can also be achieved under the action of a vertical vortex in a microfluidic chip bonded by two PDMS microchannels with slanted obstacles of exponentially increased width (Choi and Park, 2008). The proposed focusing method is suitable for larger particles and is more efficient with longer channels. Hsu et al. (2008) studied the focusing performance in microfluidics channels with the arrangement of herringbone grooves at the top of the microchannel. The focusing effect is related to channel height, channel size, groove numbers, and the ratio of groove height to channel height. By arranging herringbone grooves, particles can have a definite transverse focusing position. Chung et al. (2013b) studied the particle focusing process in microfluidic channels with the arrangement of cylindrical microstructures in the microchannel. The four equilibrium positions can be reduced to one by the vertical vortex induced by micro-obstacles, and precise focusing of a single train can be realized. Introducing micro-obstacles will result in high flow resistance, which may limit the focusing throughput and cause blockage.

When groove arrays are introduced in microchannels, a transverse pressure gradient will be generated for enhancing particle focusing. A stepped groove microchannel structure (with 30 pairs of steps) can also induce a vertical vortex (Chung et al., 2013a). The two equilibrium positions in the low *AR* rectangular channel will be gradually reduced to one due to the vertical vortex induced by the step structure in a microchannel. In stepped microfluidics, focusing performance is mainly determined by the step numbers and particle concentration. When the step groove is inclined to the straight channel, channel width will be the key element that affects focusing performance (Song and Choi, 2013). Particles focusing on high throughput (1.1 mL/min) can be realized by adopting a straight microchannel with inclined arc-shaped steps on the top side of the microchannel, as reported by Zhao et al. (2017). Zhang et al. (2013) developed a resource-saving and efficient numerical computational model and analyzed the focusing mechanism of 1–9.9 μm particles in a double-layered grooved microchannel, which provides a reliable method for predicting particle motion (Zhang et al., 2022). With the proposed numerical model, they conducted geometrical and dimensional optimization for double-layered grooved microchannels; with optimized microchannel structures, microparticles with diameter less than 1 μm can be focused (Zhang et al., 2013).

In a straight channel with an expansion–contraction cavity, a horizontal vortex at the topological cavity region and a vertical vortex downstream of the cavity will be generated due to great inertia in the fluid (Lee et al., 2011). Park et al. (2009) investigated focusing behavior in straight microchannels with topological rectangular expansion on both sides. The focusing process can be achieved by benefiting from the micro-vortex induced by channel structure and inertial lift force in the narrow pinched channel. The focusing state changes with flow rate increase: defocusing, focusing on two trains, defocusing, single-train focusing, and defocusing. Zhang et al. (2013) studied the focusing behavior in a straight microchannel with a unilateral triangular topology. The focusing efficiency is determined by particle size, and larger particles are easier to focus. According to the flow rate, the different focusing states can apply to particle focusing, separation, and trapping in microfluidic devices (Zhang et al., 2013).

For viscoelastic fluids, the streamline bends at the expansion area in the expansion–contraction channel, and the generated additional hoop stress pushes particles to the centerline (Cha et al., 2014; Jiang et al., 2021). The focusing method can improve the efficiency and throughput of elasto-inertial focusing. Yuan et al. studied the focusing behavior in straight viscoelastic microfluidics channels with a unilateral triangular topology. Combining the fluid’s elasticity, inertia, and Dean effect can achieve high-quality particle focusing. Non-Newtonian fluids are more suitable to focus particles than Newtonian fluids (Yuan et al., 2015; Yuan et al., 2017). Zhou and Papautsky (2020) summarized the progress and challenges of viscoelastic microfluidics. Recent reviews can provide more details on inertial microfluidics in contraction–expansion microchannels (Jiang et al., 2021).

Table 2 lists various particle-focusing phenomena with non-Newtonian fluid in microfluidic devices.

**TABLE 2:** Early observations of particle focusing with non-Newtonian fluid in microfluidic devices.

Structure	Microchannel	Dimension	Fluid medium	Particle (a in μm)	Dimensionless number	Reference
Straight	Square	50 × 50 μm^2^	500 ppm PEO	PS (5.9)	El (3.21–21.5)	Yang et al. (2011)
					Wi (1.61–8.04)	
	Square	80 × 80 μm^2^	0.1% w/v HA	PS (1, 3, 6, 8)	Wi (2.6–566)	Lim et al. (2014)
	Square	50 × 50 μm^2^	500 ppm PEO	PS (0.1, 0.2, 0.5, 1, 2.4)	Wi (178–533);	Young Kim et al. (2012)
		+5 × 50 μm^2^			R_c_ (0.11–0.33)	
	Square	50 × 50 μm^2^	6.6 wt% PVP	PS (6)	Wi (0.20)	Yang et al. (2012)
				RBC (8)	R_c_ (0.0007)	
	Square	50 × 50 μm^2^	2.5–50 ppm	PS (6, 10, 15)	Wi (99.4–1,491)	Kim and Kim (2016)
			λ-DNA		R_c_ (0.71–10.6)	
	Square	50 × 50 μm^2^	0.01–1 wt% PEO	PS (4.8)	Wi (0.09–25.6)	Song et al. (2016)
					R_c_ (0.1–0.8)	
	Rectangular	H = 50 μm	0.05 wt% PEO	PS (2, 5, 10)	R_c_ (0–31.71); Wi (0–97.07)	Xiang et al. (2016a)
		AR (1/3–1)	8 wt% PVP			
	Square and trapezoid	75 × 75 μm^2^	2,000 ppm PEO	PS (3, 5, 10)	∼	Raoufi et al. (2019)
	Rectangular	H = 25 μm	500–4,000 ppm PEO	PS (6.4, 10, 15)	R_c_ (0.35–30.07)	Yang et al. (2017)
		AR (1/4–1/1)			Wi (1.668–57.715)	
Spiral	Rectangular	140 × 50 μm^2^	2.0–8.0 wt% PVP	PS (10)	R_c_ (0.012–0.076)	Xiang et al. (2018)
					Wi (0.74–4.44)	
	Double spiral	30 × 4 μm^2^	0.2–0.6 wt% PEO	PS (0.05, 0.075, 0.1	Wi (0.09–0.67)	Liu et al. (2016)
				, 0.2, 0.5, 1, 2)		
	Rectangular	100 × 25 μm^2^	500–5,000 ppm PEO	PS (1.5, 5, 10)	De (2.4–18)	Lee et al. (2013)
	Rectangular	215 × 50 μm^2^	6.8 wt% PVP	PS (10)	R_c_ (0.04–9.66)	Xiang et al. (2016b)
		AR (1/4–1/2)	500 ppm PEO		Wi (0.17–47.80)	
Contraction and expansion	Square expansion	50 × 50 μm^2^	6.8 wt% PVP	PS (6)	Wi (0–5.8)	Cha et al. (2014)
		+150 × 50 μm^2^				
	Triangular expansion	100 × 50 μm^2^	500 ppm PEO	PS (3.2, 4.8, 13)	Wi (22.74–181.92); R_c_ (2.31–18.48)	Yuan et al. (2015)

### 3.4 Particle focusing in multi-staged microchannels

Multi-staged microfluidics, which typically combine multiple inertial technologies, are emerging technologies that offer better performance in terms of stability, versatility, and convenience. By combining microchannels of different focusing characteristics, the equilibrium position of particles can be controlled in multiple stages, and 3D focusing of a single train with high precision and efficiency can be achieved. Oakey et al. (2010) investigated focusing behavior in microfluidics with a straight, rectangular, and asymmetric serpentine channel. Through the synergistic effect between the multiple channel structures, the two or four roughly focused trains in straight microchannels, depending on *AR*, can be reduced to one after flow in a curved channel. The focusing behavior in the staged microfluidic device is mainly determined by the flow rate. Because the staged channel is connected in series, the focusing effect will be affected by the order of the channels. Staged microfluidics is a promising technology for high-throughput analysis and flow cytometry.

Zhang et al. (2018) designed a single-layer microfluidic chip that combined a periodic high-*AR* straight rectangular microchannel with semicircular microchannels. With the introduction of a semicircular channel, the two equilibrium positions at the top and bottom centerlines of the wide surface in rectangular channels will be reduced to one due to the stirring effect of Dean flow, and the stirring effect will be suppressed with rectangular channels. High-throughput 3D focusing can be realized with a simple channel structure (Zhang et al., 2018). Wang et al. (2015) reported high-efficiency focusing in a two-stage straight rectangular channel. The flow direction of particles is controlled by changing the hydrodynamic resistance and properly defining the channels’ length at a Y-shaped outlet in the second stage. Particle solution flows into rectangular microchannels with different aspect ratios in sequence. Particles will be focused on two central lines at the first stage with low *AR*. Then, the two equilibrium positions will shift to a single line at the high-*AR* stage. The proposed device used the advantages of being rectangular with different *AR*, which offers a convenient design for microfluidic focusing. The disadvantage may be that the device needs a long channel, which may increase the footprint. The two-staged rectangular cross-section microfluidics can also be used for particle separation due to the equilibrium position difference according to the particle size (Zhou et al., 2013). Adjusting cross-sectional shapes can be favorable for particle manipulation. Due to the size-dependent characteristics, the current focusing methods can also be applied to particle separation (Kim et al., 2018).

**FIGURE 5:**
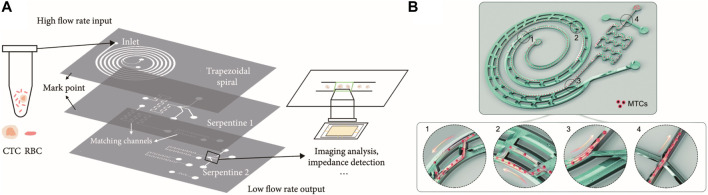
Particle focusing in the multi-staged microchannel. **(A)** 3D-stacked multistage inertial microfluidics with the trapezoidal spiral channel at the top, serpentine channel in the middle, and serpentine channel at the bottom for cell sorting (Xu et al., 2022). **(B)** Multistage inertial microfluidics for malignant tumor cell concentrators. Reproduced from Xiang and Ni (2022) with permission from the Royal Society of Chemistry.

Kim et al. (2016) reported a novel three-staged inertial focusing device that includes rectangular, triangular, and semicircular cross-section microchannels. The focusing process can be controlled according to the difference in the flow velocity profile in different cross-sections, and an efficient single-train focusing process can be achieved (Kim et al., 2016). Xu et al. (2022) proposed a 3D-stacked multistage inertial microfluidic chip cascading spiral channel with a trapezoidal cross-section and symmetrical square serpentine channels, which can achieve higher throughput in the separation and enrichment of CTCs (Figure 5A). Xiang described a low-cost multiplexed inertial microfluidic tumor cell concentrator by adopting a serial cascading asymmetric serpentine channel with the spiral channel, which will be readily integrated with other on-chip cell sorters to enhance the analysis (Xiang and Ni, 2022) (Figure 5B). More details on multi-stage microfluidics with coupled multiphysics fields can be found in Xu et al. (2021).

## 4 Conclusion and perspective

This paper reviews the working principles and channel structures of existing sheathless inertial microfluidic techniques applied to particle focusing. Inertial microfluidics, as a method of cellular sample pre-processing, offers several advantages over conventional methods by reducing the size of the necessary equipment and simplifying the complex protocols. It paves the way for a more accessible, rapid, and cost-effective way of handling biological samples, catalyzing advancements in research and clinical settings. Sheathless inertial microfluidics focusing has found various applications in the biological sciences due to its ability to precisely manipulate cells and particles in a label-free and high-throughput manner. The main applications are as follows:

Microfluidic cytometer: flow cytometry is widely used in life sciences and clinical diagnostics to characterize and analyze cells. Conventional flow cytometry uses attached probes’ scattering properties or fluorescence intensity to detect cells. Traditional flow cytometers are bulky and mechanically complex, requiring specialists to operate the equipment (Yang et al., 2018). Microfluidic cytometry has a much lower reagent/sample volume consumption and cost than conventional flow cytometry. Conventional microfluidic flow cytometers, which focus the sample stream into a narrow stream employing a surrounding sheath flow, can minimize the possibility of two or more particles simultaneously entering the optical interrogation region. Inertial microfluidics offers more freedom in multiplexing, automation, and parallelization than traditional technologies, thus adding more functionality to cell preparation, analysis, and manipulation (Yan and Yuan, 2021). With inertial microfluidics, 3D flow focusing and alignment in microfluidic channels can be achieved with a simple design, offering the potential to develop new flow cytometry systems with superior performance (Zhou et al., 2023). Microflow cytometry will become one of the most powerful technologies for rapidly analyzing cells and particles.
Cell sorting and enrichment: sorting and enrichment of target cells is a prerequisite for many biomedical applications, such as cancer detection and drug screening. Downstream analysis can be significantly simplified with sorting and enrichment. As a passive method with high manipulation efficiency, inertial microfluidics outperforms other techniques in isolating or concentrating rare cells from large background cell populations (Tang et al., 2020). Depending on the differences in inertial lift and drag forces on the particles, the equilibrium position of biological samples of different sizes within the microfluidic channel varies, and the use of inertial microfluidics such as spiral, serpentine, and constricted–expansion channels allows for the high-throughput and high-efficiency enrichment of biological samples, such as CTCs (Jeon et al., 2022; Xu et al., 2022), sperms (Feng et al., 2021), bacteria (Lu et al., 2021), and blood cells (Xiang and Ni, 2015).
Pre-processing steps for further sorting and detection: Enrichment of target cells before downstream analysis is an essential pre-processing step in many biomedical and clinical assays. Additional sheath flow is often required to focus the sample for more precisely isolating and detecting individual cells. The introduction of the sheath flow requires a sophisticated syringe pump to control the flow and keep it stable. Benefiting from the unique advantages of inertial microfluidics focusing, such as high-precision alignment and high-throughput characteristics, makes it a pre-processing step for higher precision sorting and detection. For example, inertial focusing can be used for high-precision active acoustofluidic separation (Wu et al., 2018; Peng et al., 2023). The development of integrated and efficient sorting chips can be more easily achieved by upstream inertial focusing. Inertial-focusing microfluidics for integrated cell detection has emerged as a promising trend for reducing cross-contamination and enabling single-cell unlabeled analysis (Zhou et al., 2021).

Based on a review of the existing literature, we present some prospects for sheathless inertial microfluidics focusing:

Designing an inertial microfluidic chip to achieve a specific application requires optimizing the flow rate for optimal device performance. Recently, flow-rate-insensitive spiral inertial microfluidic chips have been developed for CTC sorting applications, reducing the dependence of the device on the flow rate (Zhao et al., 2022). In the future, the development of inertial focusing microfluidic devices independent of flow rate, particle size, and other characteristics to extend applications and reduce the development cycle will be required.

At high flow rate inputs, higher pressures are typically provided. Deformation of commonly used PDMS channels causes changes in the flow profile, so implementing inertial microfluidic focusing using stiffer polymer materials will show advantages in terms of device performance and high-volume fabrication.

To enhance the integration of microfluidic chips and improve the purity and efficiency of cell separation, it is essential to combine inertial microfluidics with active separation technologies, such as coupling with multiple physical fields like acoustics, optics, and electrics. However, inertial microfluidics typically operate at high flow rates, so the flow rate mismatch with active technologies needs to be addressed. A more efficient pre-processing of biological samples can be achieved by accommodating both the high throughput of passive methods and the high precision of active methods.

In the future, with the deepening of related research, we hope that sheathless inertial microfluidic focusing can be integrated into biological sample sampling, separation, and detection and can be efficiently and practically used in POCT health monitoring.

## References

[B1] Amani A. Shamloo A. Vatani P. Ebrahimi S. (2022). Particles focusing and separation by a novel inertial microfluidic device: divergent serpentine microchannel. Ind. Eng. Chem. Res. 61, 14324–14333. 10.1021/acs.iecr.2c02451

[B2] Amini H. Lee W. Di Carlo D. (2014). Inertial microfluidic physics. Lab. Chip 14, 2739–2761. 10.1039/c4lc00128a 24914632

[B3] Asghari M. Cao X. Mateescu B. van Leeuwen D. Stavrakis S. DeMello A. (2019). Oscillatory viscoelastic microfluidics for efficient focusing and separation of nanoscale species. bioRxiv. 10.1101/668301 31794192

[B4] Bai J.-J. Zhang X. Wei X. Wang Y. Du C. Wang Z.-J. (2023). Dean-flow-coupled elasto-inertial focusing accelerates exosome purification to facilitate single vesicle profiling. Anal. Chem. 95, 2523–2531. 10.1021/acs.analchem.2c04898 36657481

[B5] Bayareh M. (2020). An updated review on particle separation in passive microfluidic devices. Chem. Eng. Process. - Process Intensif. 153, 107984. 10.1016/j.cep.2020.107984

[B6] Bhagat A. A. S. Kuntaegowdanahalli S. S. Papautsky I. (2008). Continuous particle separation in spiral microchannels using dean flows and differential migration. Lab. Chip 8, 1906–1914. 10.1039/b807107a 18941692

[B7] Bhagat A. A. S. Kuntaegowdanahalli S. S. Papautsky I. (2009). Inertial microfluidics for continuous particle filtration and extraction. Microfluid. Nanofluidics 7, 217–226. 10.1007/s10404-008-0377-2

[B8] Caserta S. D’Avino G. Greco F. Guido S. Maffettone P. L. (2011). Migration of a sphere in a viscoelastic fluid under planar shear flow: experiments and numerical predictions. Soft Matter 7, 1100–1106. 10.1039/c0sm00640h

[B9] Cha H. Amiri H. A. Moshafi S. Karimi A. Nikkhah A. Chen X. (2023a). Effects of obstacles on inertial focusing and separation in sinusoidal channels: an experimental and numerical study. Chem. Eng. Sci. 276, 118826. 10.1016/j.ces.2023.118826

[B10] Cha H. Dai Y. Hansen H. H. W. B. Ouyang L. Chen X. Kang X. (2023b). Asymmetrical obstacles enable unilateral inertial focusing and separation in sinusoidal microchannel. Cyborg Bionic Syst. 4, 0036. 10.34133/cbsystems.0036 37342212 PMC10278993

[B11] Cha H. Fallahi H. Dai Y. Yadav S. Hettiarachchi S. McNamee A. (2022). Tuning particle inertial separation in sinusoidal channels by embedding periodic obstacle microstructures. Lab. Chip 22, 2789–2800. 10.1039/D2LC00197G 35587546

[B12] Cha S. Kang K. You J. B. Im S. G. Kim Y. Kim J. M. (2014). Hoop stress-assisted three-dimensional particle focusing under viscoelastic flow. Rheol. Acta 53, 927–933. 10.1007/s00397-014-0808-9

[B13] Chiu Y. J. Cho S. H. Mei Z. Lien V. Wu T. F. Lo Y. H. (2013). Universally applicable three-dimensional hydrodynamic microfluidic flow focusing. Lab. Chip 13, 1803. 10.1039/c3lc41202d 23493956 PMC3654829

[B14] Choi S. Park J. K. (2007). Continuous hydrophoretic separation and sizing of microparticles using slanted obstacles in a microchannel. Lab. Chip 7, 890–897. 10.1039/b701227f 17594009

[B15] Choi S. Park J. K. (2008). Sheathless hydrophoretic particle focusing in a microchannel with exponentially increasing obstacle arrays. Anal. Chem. 80, 3035–3039. 10.1021/ac8001319 18355090

[B16] Chung A. J. Gossett D. R. Di Carlo D. (2013a). Three dimensional, sheathless, and high-throughput microparticle inertial focusing through geometry-induced secondary flows. Small 9, 685–690. 10.1002/smll.201202413 23143944

[B17] Chung A. J. Pulido D. Oka J. C. Amini H. Masaeli M. Di Carlo D. (2013b). Microstructure-induced helical vortices allow single-stream and long-term inertial focusing. Lab. Chip 13, 2942–2949. 10.1039/c3lc41227j 23665981

[B18] Ciftlik A. T. Ettori M. Gijs M. A. M. (2013). High throughput-per-footprint inertial focusing. Small 9, 2764–2773. 10.1002/smll.201201770 23420756

[B19] Cruz J. Graells T. Walldén M. Hjort K. (2019). Inertial focusing with sub-micron resolution for separation of bacteria. Lab. Chip 19, 1257–1266. 10.1039/C9LC00080A 30821308

[B20] Del Giudice F. Romeo G. D’Avino G. Greco F. Netti P. A. Maffettone P. L. (2013). Particle alignment in a viscoelastic liquid flowing in a square-shaped microchannel. Lab. Chip 13, 4263–4271. 10.1039/c3lc50679g 24056525

[B21] Di Carlo D. (2009). Inertial microfluidics. Lab. Chip 9, 3038. 10.1039/b912547g 19823716

[B22] Di Carlo D. Edd J. F. Irimia D. Tompkins R. G. Toner M. (2008). Equilibrium separation and filtration of particles using differential inertial focusing. Anal. Chem. 80, 2204–2211. 10.1021/ac702283m 18275222

[B23] Di Carlo D. Irimia D. Tompkins R. G. Toner M. (2007). Continuous inertial focusing, ordering, and separation of particles in microchannels. Proc. Natl. Acad. Sci. 104, 18892–18897. 10.1073/pnas.0704958104 18025477 PMC2141878

[B24] Ebrahimi S. Alishiri M. Shamloo A. Pishbin E. Hemmati P. Seifi S. (2023). Optimizing the design of a serpentine microchannel based on particles focusing and separation: a numerical study with experimental validation. Sensors Actuators A Phys. 358, 114432. 10.1016/j.sna.2023.114432

[B25] Fan L. L. Han Y. He X. K. Zhao L. Zhe J. (2014). High-throughput, single-stream microparticle focusing using a microchannel with asymmetric sharp corners. Microfluid. Nanofluidics 17, 639–646. 10.1007/s10404-014-1344-8

[B26] Feng H. Jafek A. Samuel R. Hotaling J. Jenkins T. G. Aston K. I. (2021). High efficiency rare sperm separation from biopsy samples in an inertial focusing device. Analyst 146, 3368–3377. 10.1039/D1AN00480H 33871507

[B27] Feng H. Jafek A. R. Wang B. Brady H. Magda J. J. Gale B. K. (2022). Viscoelastic particle focusing and separation in a spiral channel. Micromachines 13, 361. 10.3390/mi13030361 35334653 PMC8954746

[B28] Gao H. Zhou J. Naderi M. M. Peng Z. Papautsky I. (2023). Evolution of focused streams for viscoelastic flow in spiral microchannels. Microsystems Nanoeng. 9, 73. 10.1038/s41378-023-00520-4 PMC10241945 37288322

[B29] Gossett D. R. Di Carlo D. (2009). Particle focusing mechanisms in curving confined flows. Anal. Chem. 81, 8459–8465. 10.1021/ac901306y 19761190

[B30] Gou Y. Zhang S. Sun C. Wang P. You Z. Yalikun Y. (2020). Sheathless inertial focusing chip combining a spiral channel with periodic expansion structures for efficient and stable particle sorting. Anal. Chem. 92, 1833–1841. 10.1021/acs.analchem.9b03692 31858787

[B31] Guan G. Wu L. Bhagat A. A. Li Z. Chen P. C. Y. Chao S. (2013). Spiral microchannel with rectangular and trapezoidal cross-sections for size based particle separation. Sci. Rep. 3, 1475. 10.1038/srep01475 23502529 PMC3600595

[B32] Hettiarachchi S. Cha H. Ouyang L. Mudugamuwa A. An H. Kijanka G. (2023). Recent microfluidic advances in submicron to nanoparticle manipulation and separation. Lab. Chip 23, 982–1010. 10.1039/D2LC00793B 36367456

[B33] Hsu C. H. Di Carlo D. Chen C. Irimia D. Toner M. (2008). Microvortex for focusing, guiding and sorting of particles. Lab. Chip 8, 2128–2134. 10.1039/b813434k 19023476 PMC4142319

[B34] Hur S. C. Henderson-Maclennan N. K. McCabe E. R. B. Di Carlo D. (2011). Deformability-based cell classification and enrichment using inertial microfluidics. Lab. Chip 11, 912–920. 10.1039/c0lc00595a 21271000

[B35] Jeon H. Kwon T. Yoon J. Han J. (2022). Engineering a deformation-free plastic spiral inertial microfluidic system for CHO cell clarification in biomanufacturing. Lab. Chip 22, 272–285. 10.1039/D1LC00995H 34931631

[B36] Jia Z. Wu J. Wu X. Yuan Q. Chan Y. Liu B. (2023). Size-tunable elasto-inertial sorting of *<i>Haematococcus pluvialis*</i> in the ultrastretchable microchannel. Anal. Chem. 95, 13338–13345. 10.1021/acs.analchem.3c02648 37585740

[B37] Jiang D. Ni C. Tang W. Huang D. Xiang N. (2021). Inertial microfluidics in contraction–expansion microchannels: a review. Biomicrofluidics 15, 041501. 10.1063/5.0058732 34262632 PMC8254650

[B38] Kim B. Kim J. M. (2016). Elasto-inertial particle focusing under the viscoelastic flow of DNA solution in a square channel. Biomicrofluidics 10, 024111. 10.1063/1.4944628 27051468 PMC4808065

[B39] Kim J. Choi Y. Lee W. (2022a). Alteration of inertial focusing positions in triangular channels using flexible PDMS microfluidics. BioChip J. 16, 342–350. 10.1007/s13206-022-00062-3

[B40] Kim J. Lee J. Wu C. Nam S. Di Carlo D. Lee W. (2016). Inertial focusing in non-rectangular cross-section microchannels and manipulation of accessible focusing positions. Lab. Chip 16, 992–1001. 10.1039/C5LC01100K 26853995

[B41] Kim J. A. Lee J. R. Je T. J. Jeon E. C. Lee W. (2018). Size-dependent inertial focusing position shift and particle separations in triangular microchannels. Anal. Chem. 90, 1827–1835. 10.1021/acs.analchem.7b03851 29271639

[B42] Kim U. Kwon J.-Y. Kim T. Cho Y. (2022b). Particle focusing in a straight microchannel with non-rectangular cross-section. Micromachines 13, 151. 10.3390/mi13020151 35208276 PMC8875687

[B43] Kumar T. Ramachandraiah H. Iyengar S. N. Banerjee I. Mårtensson G. Russom A. (2021). High throughput viscoelastic particle focusing and separation in spiral microchannels. Sci. Rep. 11, 8467. 10.1038/s41598-021-88047-4 33875755 PMC8055915

[B44] Kuntaegowdanahalli S. S. Bhagat A. A. S. Kumar G. Papautsky I. (2009). Inertial microfluidics for continuous particle separation in spiral microchannels. Lab. Chip 9, 2973. 10.1039/b908271a 19789752

[B45] Lee D. J. Brenner H. Youn J. R. Song Y. S. (2013). Multiplex particle focusing via hydrodynamic force in Viscoelastic Fluids. Sci. Rep. 3, 3258–3310. 10.1038/srep03258 24247252 PMC3832872

[B46] Lee M. G. Choi S. Kim H.-J. Lim H. K. Kim J.-H. Huh N. (2011). Inertial blood plasma separation in a contraction–expansion array microchannel. Appl. Phys. Lett. 98, 253702. 10.1063/1.3601745

[B48] Leshansky A. M. Bransky A. Korin N. Dinnar U. (2007). Tunable nonlinear viscoelastic “focusing” in a microfluidic device. Phys. Rev. Lett. 98, 234501. 10.1103/PhysRevLett.98.234501 17677908

[B49] Lim E. J. Ober T. J. Edd J. F. Desai S. P. Neal D. Bong K. W. (2014). Inertio-elastic focusing of bioparticles in microchannels at high throughput. Nat. Commun. 5, 4120. 10.1038/ncomms5120 24939508 PMC4476514

[B50] Liu C. Ding B. Xue C. Tian Y. Hu G. Sun J. (2016). Sheathless focusing and separation of diverse nanoparticles in viscoelastic solutions with minimized shear thinning. Anal. Chem. 88, 12547–12553. 10.1021/acs.analchem.6b04564 28193038

[B51] Liu C. Xue C. Chen X. Shan L. Tian Y. Hu G. (2015). Size-based separation of particles and cells utilizing viscoelastic effects in straight microchannels. Anal. Chem. 87, 6041–6048. 10.1021/acs.analchem.5b00516 25989347

[B52] Liu Y. Shen H. Yang X. Kang S. Cai L. Tian T. (2023). Recent progress in microfluidic biosensors with different driving forces. Trac. Trends Anal. Chem. 158, 116894. 10.1016/j.trac.2022.116894

[B53] Lu M. Ozcelik A. Grigsby C. L. Zhao Y. Guo F. Leong K. W. (2016). Microfluidic hydrodynamic focusing for synthesis of nanomaterials. Nano Today 11, 778–792. 10.1016/j.nantod.2016.10.006 30337950 PMC6191180

[B54] Lu X. Chow J. J. M. Koo S. H. Jiang B. Tan T. Y. Yang D. (2021). Sheathless and high-throughput elasto-inertial bacterial sorting for enhancing molecular diagnosis of bloodstream infection. Lab. Chip 21, 2163–2177. 10.1039/D1LC00085C 33899072

[B55] Lu X. Chow J. J. M. Koo S. H. Tan T. Y. Jiang B. Ai Y. (2020). Enhanced molecular diagnosis of bloodstream Candida infection with size-based inertial sorting at submicron resolution. Anal. Chem. 92, 15579–15586. 10.1021/acs.analchem.0c03718 33191733

[B56] Lu X. Liu C. Hu G. Xuan X. (2017). Particle manipulations in non-Newtonian microfluidics: a review. J. Colloid Interface Sci. 500, 182–201. 10.1016/j.jcis.2017.04.019 28412635

[B57] Mao X. Waldeisen J. R. Huang T. J. (2007). “Microfluidic drifting” - implementing three-dimensional hydrodynamic focusing with a single-layer planar microfluidic device. Lab. Chip 7, 1260–1262. 10.1039/b711155j 17896008

[B58] Martel J. M. Toner M. (2012). Inertial focusing dynamics in spiral microchannels. Phys. Fluids 24, 032001. 10.1063/1.3681228 PMC3311666 22454556

[B59] Martel J. M. Toner M. (2013). Particle focusing in curved microfluidic channels. Sci. Rep. 3, 3340. 10.1038/srep03340

[B60] Masaeli M. Sollier E. Amini H. Mao W. Camacho K. Doshi N. (2012). Continuous inertial focusing and separation of particles by shape. Phys. Rev. X 2, 031017–31113. 10.1103/PhysRevX.2.031017

[B61] Mashhadian A. Shamloo A. (2019). Inertial microfluidics: a method for fast prediction of focusing pattern of particles in the cross section of the channel. Anal. Chim. Acta 1083, 137–149. 10.1016/j.aca.2019.06.057 31493804

[B62] Matas J. Morris J. Guazzelli E. (2004). Lateral forces on a sphere. Oil Gas. Sci. Technol. 59, 59–70. 10.2516/ogst:2004006

[B63] Moloudi R. Oh S. Yang C. Ebrahimi Warkiani M. Naing M. W. (2018). Inertial particle focusing dynamics in a trapezoidal straight microchannel: application to particle filtration. Microfluid. Nanofluidics 22, 33–14. 10.1007/s10404-018-2045-5

[B64] Mukherjee P. Wang X. Zhou J. Papautsky I. (2019). Single stream inertial focusing in low aspect-ratio triangular microchannels. Lab. Chip 19, 147–157. 10.1039/c8lc00973b 30488049

[B65] Mutlu B. R. Edd J. F. Toner M. (2018). Oscillatory inertial focusing in infinite microchannels. Proc. Natl. Acad. Sci. 115, 7682–7687. 10.1073/pnas.1721420115 29991599 PMC6065022

[B66] Nagrath S. Sequist L. V. Maheswaran S. Bell D. W. Irimia D. Ulkus L. (2007). Isolation of rare circulating tumour cells in cancer patients by microchip technology. Nature 450, 1235–1239. 10.1038/nature06385 18097410 PMC3090667

[B67] Nam J. Lim H. Kim D. Jung H. Shin S. (2012). Continuous separation of microparticles in a microfluidic channel via the elasto-inertial effect of non-Newtonian fluid. Lab. Chip 12, 1347–1354. 10.1039/c2lc21304d 22334376

[B68] Narayanamurthy V. Jeroish Z. E. Bhuvaneshwari K. S. Bayat P. Premkumar R. Samsuri F. (2020). Advances in passively driven microfluidics and lab-on-chip devices: a comprehensive literature review and patent analysis. RSC Adv. 10, 11652–11680. 10.1039/D0RA00263A 35496619 PMC9050787

[B69] Ni C. Zhou Z. Zhu Z. Jiang D. Xiang N. (2022). Controllable size-independent three-dimensional inertial focusing in high-aspect-ratio asymmetric serpentine microchannels. Anal. Chem. 94, 15639–15647. 10.1021/acs.analchem.2c02361 36315448

[B70] Nilsson J. Evander M. Hammarström B. Laurell T. (2009). Review of cell and particle trapping in microfluidic systems. Anal. Chim. Acta 649, 141–157. 10.1016/j.aca.2009.07.017 19699390

[B71] Oakey J. Applegate R. W. Arellano E. Carlo D. Graves S. W. Toner M. (2010). Particle focusing in staged inertial microfluidic devices for flow cytometry. Anal. Chem. 82, 3862–3867. 10.1021/ac100387b 20373755 PMC3136802

[B72] Ookawara S. Higashi R. Street D. Ogawa K. (2004). Feasibility study on concentration of slurry and classification of contained particles by microchannel. Chem. Eng. J. 101, 171–178. 10.1016/j.cej.2003.11.008

[B73] Özbey A. Karimzadehkhouei M. Akgönül S. Gozuacik D. Koşar A. (2016). Inertial focusing of microparticles in curvilinear microchannels. Sci. Rep. 6, 38809–38811. 10.1038/srep38809 27991494 PMC5171716

[B74] Paiè P. Bragheri F. Di Carlo D. Osellame R. (2017). Particle focusing by 3D inertial microfluidics. Microsystems Nanoeng. 3, 17027–17028. 10.1038/micronano.2017.27 PMC6444990 31057868

[B75] Park J. S. Song S. H. Jung H. (2009). Continuous focusing of microparticles using inertial lift force and vorticity via multi-orifice microfluidic channels. Lab. Chip 9, 939–948. 10.1039/b813952k 19294305

[B76] Pathak J. A. Ross D. Migler K. B. (2004). Elastic flow instability, curved streamlines, and mixing in microfluidic flows. Phys. Fluids 16, 4028–4034. 10.1063/1.1792011

[B77] Peng T. Qiang J. Yuan S. (2023). Investigation on a cascaded inertial and acoustic microfluidic device for sheathless and label-free separation of circulating tumor cells. Phys. Fluids 35. 10.1063/5.0160391

[B78] Raoufi M. A. Mashhadian A. Niazmand H. Asadnia M. Razmjou A. Warkiani M. E. (2019). Experimental and numerical study of elasto-inertial focusing in straight channels. Biomicrofluidics 13, 034103. 10.1063/1.5093345 31123535 PMC6509046

[B79] Razavi Bazaz S. Mihandust A. Salomon R. Joushani H. A. N. Li W. Amiri H. (2022). Zigzag microchannel for rigid inertial separation and enrichment (Z-RISE) of cells and particles. Lab. Chip 22, 4093–4109. 10.1039/D2LC00290F 36102894

[B80] Reece A. E. Oakeya J. (2016). Long-range forces affecting equilibrium inertial focusing behavior in straight high aspect ratio microfluidic channels. Phys. Fluids 28, 043303. 10.1063/1.4946829 PMC4851623 27190494

[B81] Rodd L. E. Cooper-White J. J. Boger D. V. McKinley G. H. (2007). Role of the elasticity number in the entry flow of dilute polymer solutions in micro-fabricated contraction geometries. J. Nonnewt. Fluid Mech. 143, 170–191. 10.1016/j.jnnfm.2007.02.006

[B82] Rodd L. E. Scott T. P. Boger D. V. Cooper-White J. J. McKinley G. H. (2005). The inertio-elastic planar entry flow of low-viscosity elastic fluids in micro-fabricated geometries. J. Nonnewt. Fluid Mech. 129, 1–22. 10.1016/j.jnnfm.2005.04.006

[B83] Rubinow S. I. Keller J. B. (1961). The transverse force on a spinning sphere moving in a viscous fluid. J. Fluid Mech. 11, 447. 10.1017/S0022112061000640

[B84] Rufo J. Cai F. Friend J. Wiklund M. Huang T. J. (2022). Acoustofluidics for biomedical applications. Nat. Rev. Methods Prim. 2, 30. 10.1038/s43586-022-00109-7

[B85] Russom A. Gupta A. K. Nagrath S. Carlo D. Toner M. (2009). Differential inertial focusing of particles in curved low-aspect-ratio microchannels. New J. Phys. 11, 075025. 10.1088/1367-2630/11/7/075025 PMC2942776 20862272

[B86] Saffman P. G. (1965). The lift on a small sphere in a slow shear flow. J. Fluid Mech. 22, 385–400. 10.1017/S0022112065000824

[B87] Segré G. Silberberg A. (1961). Radial particle displacements in Poiseuille flow of suspensions. Nature 189, 209–210. 10.1038/189209a0

[B88] Seo K. W. Byeon H. J. Huh H. K. Lee S. J. (2014). Particle migration and single-line particle focusing in microscale pipe flow of viscoelastic fluids. RSC Adv. 4, 3512–3520. 10.1039/c3ra43522a

[B89] Shamloo A. Mashhadian A. (2018). Inertial particle focusing in serpentine channels on a centrifugal platform. Phys. Fluids 30, 2621. 10.1063/1.5002621

[B90] Shen S. Tian C. Li T. Xu J. Chen S. W. Tu Q. (2017). Spiral microchannel with ordered micro-obstacles for continuous and highly-efficient particle separation. Lab. Chip 17, 3578–3591. 10.1039/c7lc00691h 28975177

[B91] Shi R. (2023). Numerical simulation of inertial microfluidics: a review. Eng. Appl. Comput. Fluid Mech. 17. 10.1080/19942060.2023.2177350

[B92] Song H. Y. Lee S. H. Salehiyan R. Hyun K. (2016). Relationship between particle focusing and dimensionless numbers in elasto-inertial focusing. Rheol. Acta 55, 889–900. 10.1007/s00397-016-0962-3

[B93] Song S. Choi S. (2013). Design rules for size-based cell sorting and sheathless cell focusing by hydrophoresis. J. Chromatogr. A 1302, 191–196. 10.1016/j.chroma.2013.06.030 23838306

[B94] Stan C. A. Ellerbee A. K. Guglielmini L. Stone H. A. Whitesides G. M. (2013). The magnitude of lift forces acting on drops and bubbles in liquids flowing inside microchannels. Lab. Chip 13, 365–376. 10.1039/C2LC41035D 23212283

[B95] Su J. Chen X. Zhu Y. Hu G. (2021). Machine learning assisted fast prediction of inertial lift in microchannels. Lab. Chip 21, 2544–2556. 10.1039/D1LC00225B 33998624

[B96] Su J. Zheng X. Hu G. (2023). New explicit formula for inertial lift in confined flows. Phys. Fluids 35. 10.1063/5.0168474

[B97] Tang W. Zhu S. Jiang D. Zhu L. Yang J. Xiang N. (2020). Channel innovations for inertial microfluidics. Lab. Chip 20, 3485–3502. 10.1039/D0LC00714E 32910129

[B98] Wang C. Sun S. Chen Y. Cheng Z. Li Y. Jia L. (2018). Inertial particle focusing and spacing control in microfluidic devices. Microfluid. Nanofluidics 22, 25. 10.1007/s10404-018-2035-7

[B99] Wang L. Dandy D. S. (2017). High-throughput inertial focusing of micrometer- and sub-micrometer-sized particles separation. Adv. Sci. 4, 1700153. 10.1002/advs.201700153 PMC5644225 29051857

[B100] Wang X. Zandi M. Ho C. C. Kaval N. Papautsky I. (2015). Single stream inertial focusing in a straight microchannel. Lab. Chip 15, 1812–1821. 10.1039/c4lc01462f 25761900 PMC4388233

[B101] Whitesides G. M. (2006). The origins and the future of microfluidics. Nature 442, 368–373. 10.1038/nature05058 16871203

[B102] Won Seo K. Ran Ha Y. Joon Lee S. (2014). Vertical focusing and cell ordering in a microchannel via viscoelasticity: applications for cell monitoring using a digital holographic microscopy. Appl. Phys. Lett. 104, 213702. 10.1063/1.4880615

[B103] Wu M. Chen K. Yang S. Wang Z. Huang P.-H. Mai J. (2018). High-throughput cell focusing and separation via acoustofluidic tweezers. Lab. Chip 18, 3003–3010. 10.1039/C8LC00434J 30131991 PMC6203445

[B104] Xiang N. Chen K. Sun D. Wang S. Yi H. Ni Z. (2013a). Quantitative characterization of the focusing process and dynamic behavior of differently sized microparticles in a spiral microchannel. Microfluid. Nanofluidics 14, 89–99. 10.1007/s10404-012-1025-4

[B105] Xiang N. Dai Q. Ni Z. (2016a). Multi-train elasto-inertial particle focusing in straight microfluidic channels. Appl. Phys. Lett. 109, 134101. 10.1063/1.4963294

[B106] Xiang N. Ni Z. (2015). High-throughput blood cell focusing and plasma isolation using spiral inertial microfluidic devices. Biomed. Microdevices 17, 110. 10.1007/s10544-015-0018-y 26553099

[B107] Xiang N. Ni Z. (2022). High-throughput concentration of rare malignant tumor cells from large-volume effusions by multistage inertial microfluidics. Lab. Chip 22, 757–767. 10.1039/D1LC00944C 35050294

[B108] Xiang N. Ni Z. Yi H. (2018). Concentration-controlled particle focusing in spiral elasto-inertial microfluidic devices. Electrophoresis 39, 417–424. 10.1002/elps.201700150 28990196

[B109] Xiang N. Shi Z. Tang W. Huang D. Zhang X. Ni Z. (2015). Improved understanding of particle migration modes in spiral inertial microfluidic devices. RSC Adv. 5, 77264–77273. 10.1039/c5ra13292d

[B110] Xiang N. Yi H. Chen K. Sun D. Jiang D. Dai Q. (2013b). High-throughput inertial particle focusing in a curved microchannel: insights into the flow-rate regulation mechanism and process model. Biomicrofluidics 7, 44116–44210. 10.1063/1.4818445 24404049 PMC3751952

[B111] Xiang N. Zhang X. Dai Q. Cheng J. Chen K. Ni Z. (2016b). Fundamentals of elasto-inertial particle focusing in curved microfluidic channels. Lab. Chip 16, 2626–2635. 10.1039/c6lc00376a 27300118

[B112] Xu X. Huang X. Sun J. Chen J. Wu G. Yao Y. (2022). 3D-Stacked multistage inertial microfluidic chip for high-throughput enrichment of circulating tumor cells. Cyborg Bionic Syst. 2022, 9829287. 10.34133/2022/9829287 PMC11030111 38645277

[B113] Xu X. Huang X. Sun J. Wang R. Yao J. Han W. (2021). Recent progress of inertial microfluidic-based cell separation. Analyst 146, 7070–7086. 10.1039/D1AN01160J 34761757

[B114] Xuan X. Zhu J. Church C. (2010). Particle focusing in microfluidic devices. Microfluid. Nanofluidics 9, 1–16. 10.1007/s10404-010-0602-7

[B115] Yan S. Yuan D. (2021). Continuous microfluidic 3D focusing enabling microflow cytometry for single-cell analysis. Talanta 221, 121401. 10.1016/j.talanta.2020.121401 33076055

[B116] Yang R.-J. Fu L.-M. Hou H.-H. (2018). Review and perspectives on microfluidic flow cytometers. Sensors Actuators B Chem. 266, 26–45. 10.1016/j.snb.2018.03.091

[B117] Yang S. Kim J. Y. Lee S. J. Lee S. S. Kim J. M. (2011). Sheathless elasto-inertial particle focusing and continuous separation in a straight rectangular microchannel. Lab. Chip 11, 266–273. 10.1039/c0lc00102c 20976348

[B118] Yang S. Lee S. S. Ahn S. W. Kang K. Shim W. Lee G. (2012). Deformability-selective particle entrainment and separation in a rectangular microchannel using medium viscoelasticity. Soft Matter 8, 5011–5019. 10.1039/c2sm07469a

[B119] Yang S. H. Lee D. J. Youn J. R. Song Y. S. (2017). Multiple-line particle focusing under viscoelastic flow in a microfluidic device. Anal. Chem. 89, 3639–3647. 10.1021/acs.analchem.6b05052 28225617

[B120] Yang X. Gong C. Zhang C. Wang Y. Yan G. Wei L. (2022). Fiber optofluidic microlasers: structures, characteristics, and applications. Laser Phot. Rev. 16, 171. 10.1002/lpor.202100171

[B121] Young Kim J. Won Ahn S. Sik Lee S. Min Kim J. (2012). Lateral migration and focusing of colloidal particles and DNA molecules under viscoelastic flow. Lab. Chip 12, 2807–2814. 10.1039/c2lc40147a 22776909

[B122] Yuan D. Sluyter R. Zhao Q. Tang S. Yan S. Yun G. (2019). Dean-flow-coupled elasto-inertial particle and cell focusing in symmetric serpentine microchannels. Microfluid. Nanofluidics 23, 41. 10.1007/s10404-019-2204-3

[B123] Yuan D. Tan S. H. Zhao Q. Yan S. Sluyter R. Nguyen N. T. (2017). Sheathless Dean-flow-coupled elasto-inertial particle focusing and separation in viscoelastic fluid. RSC Adv. 7, 3461–3469. 10.1039/C6RA25328H

[B124] Yuan D. Zhang J. Yan S. Pan C. Alici G. Nguyen N. T. (2015). Dean-flow-coupled elasto-inertial three-dimensional particle focusing under viscoelastic flow in a straight channel with asymmetrical expansion–contraction cavity arrays. Biomicrofluidics 9, 044108. 10.1063/1.4927494 26339309 PMC4522007

[B125] Yuan D. Zhao Q. Yan S. Tang S. Y. Alici G. Zhang J. (2018). Recent progress of particle migration in viscoelastic fluids. Lab. Chip 18, 551–567. 10.1039/c7lc01076a 29340388

[B126] Zeng L. Balachandar S. Fischer P. (2005). Wall-induced forces on a rigid sphere at finite Reynolds number. J. Fluid Mech. 536, 1–25. 10.1017/S0022112005004738

[B145] Zhang B. R. Fan Y. Wu S. Wan W. Zhao W. Zhao Q. (2022). “Investigation of particle manipulation mechanism and size sorting strategy in a double‐layered microchannel,” in Lab on a Chip 22.23, 4556–4573. 10.1039/d2lc00822j 36321548

[B127] Zhang J. Li M. Li W. H. Alici G. (2013). Inertial focusing in a straight channel with asymmetrical expansion–contraction cavity arrays using two secondary flows. J. Micromechanics Microengineering 23, 085023. 10.1088/0960-1317/23/8/085023

[B128] Zhang J. Li W. Li M. Alici G. Nguyen N.-T. (2014). Particle inertial focusing and its mechanism in a serpentine microchannel. Microfluid. Nanofluidics 17, 305–316. 10.1007/s10404-013-1306-6

[B129] Zhang J. Yan S. Yuan D. Alici G. Nguyen N. T. Ebrahimi Warkiani M. (2016). Fundamentals and applications of inertial microfluidics: a review. Lab. Chip 16, 10–34. 10.1039/c5lc01159k 26584257

[B130] Zhang J. Yuan D. Zhao Q. Teo A. J. T. Yan S. Ooi C. H. (2019). Fundamentals of differential particle inertial focusing in symmetric sinusoidal microchannels. Anal. Chem. 91, 4077–4084. 10.1021/acs.analchem.8b05712 30669838

[B131] Zhang T. Hong Z.-Y. Tang S.-Y. Li W. Inglis D. W. Hosokawa Y. (2020). Focusing of sub-micrometer particles in microfluidic devices. Lab. Chip 20, 35–53. 10.1039/C9LC00785G 31720655

[B132] Zhang Y. Zhang J. Tang F. Li W. Wang X. (2018). Design of a single-layer microchannel for continuous sheathless single-stream particle inertial focusing. Anal. Chem. 90, 1786–1794. 10.1021/acs.analchem.7b03756 29297226

[B133] Zhao L. Gao M. Niu Y. Wang J. Shen S. (2022). Flow-rate and particle-size insensitive inertial focusing in dimension-confined ultra-low aspect ratio spiral microchannel. Sensors Actuators B Chem. 369, 132284. 10.1016/j.snb.2022.132284

[B134] Zhao Q. Yuan D. Zhang J. Li W. (2020). A review of secondary flow in inertial microfluidics. Micromachines 11, 461. 10.3390/mi11050461 32354106 PMC7280964

[B135] Zhao Q. Zhang J. Yan S. Yuan D. Du H. Alici G. (2017). High-throughput sheathless and three-dimensional microparticle focusing using a microchannel with arc-shaped groove arrays. Sci. Rep. 7, 41153–41211. 10.1038/srep41153 28112225 PMC5253733

[B136] Zhou J. Giridhar P. V. Kasper S. Papautsky I. (2013). Modulation of aspect ratio for complete separation in an inertial microfluidic channel. Lab. Chip 13, 1919. 10.1039/c3lc50101a 23529341

[B137] Zhou J. Papautsky I. (2013). Fundamentals of inertial focusing in microchannels. Lab. Chip 13, 1121–1132. 10.1039/c2lc41248a 23353899

[B138] Zhou J. Papautsky I. (2020). Viscoelastic microfluidics: progress and challenges. Microsystems Nanoeng. 6, 113. 10.1038/s41378-020-00218-x PMC8433399 34567720

[B139] Zhou Y. Ma Z. Ai Y. (2018). Sheathless inertial cell focusing and sorting with serial reverse wavy channel structures. Microsystems Nanoeng. 4, 5. 10.1038/s41378-018-0005-6 PMC6220157 31057895

[B140] Zhou Z. Chen Y. Zhu S. Liu L. Ni Z. Xiang N. (2021). Inertial microfluidics for high-throughput cell analysis and detection: a review. Analyst 146, 6064–6083. 10.1039/D1AN00983D 34490431

[B141] Zhou Z. Ni C. Zhu Z. Chen Y. Ni Z. Xiang N. (2023). High-throughput adjustable deformability cytometry utilizing elasto-inertial focusing and virtual fluidic channel. Lab. Chip 23, 4528–4539. 10.1039/D3LC00591G 37766593

[B142] Zhu P. Wang L. (2017). Passive and active droplet generation with microfluidics: a review. Lab. Chip 17, 34–75. 10.1039/C6LC01018K 27841886

[B143] Zhu Z. Wu D. Li S. Han Y. Xiang N. Wang C. (2021). A polymer-film inertial microfluidic sorter fabricated by jigsaw puzzle method for precise size-based cell separation. Anal. Chim. Acta 1143, 306–314. 10.1016/j.aca.2020.11.001 33384126

